# A systematic review and meta-analysis of circulating adhesion molecules in rheumatoid arthritis

**DOI:** 10.1007/s00011-023-01837-6

**Published:** 2024-01-19

**Authors:** Arduino A. Mangoni, Angelo Zinellu

**Affiliations:** 1 Discipline of Clinical Pharmacology, College of Medicine and Public Health, Flinders University, Adelaide, Australia; 2 Department of Clinical Pharmacology, Flinders Medical Centre, Southern Adelaide Local Health Network, Adelaide, Australia; 3 Department of Biomedical Sciences, University of Sassari, Sassari, Italy; 4 Department of Clinical Pharmacology, College of Medicine and Public Health, Flinders University and Flinders Medical Centre, Bedford Park, SA 5042 Australia

**Keywords:** Cell adhesion molecules, Selectins, Rheumatoid arthritis, Atherosclerosis, Cardiovascular disease, Biomarkers

## Abstract

**Background:**

The availability of robust biomarkers of endothelial activation might enhance the identification of subclinical atherosclerosis in rheumatoid arthritis (RA). We investigated this issue by conducting a systematic review and meta-analysis of cell adhesion molecules in RA patients.

**Methods:**

We searched electronic databases from inception to 31 July 2023 for case–control studies assessing the circulating concentrations of immunoglobulin-like adhesion molecules (vascular cell, VCAM-1, intercellular, ICAM-1, and platelet endothelial cell, PECAM-1, adhesion molecule-1) and selectins (E, L, and P selectin) in RA patients and healthy controls. Risk of bias and certainty of evidence were assessed using the JBI checklist and GRADE, respectively.

**Results:**

In 39 studies, compared to controls, RA patients had significantly higher concentrations of ICAM-1 (standard mean difference, SMD = 0.81, 95% CI 0.62–1.00, *p* < 0.001; *I*^2^ = 83.0%, *p* < 0.001), VCAM-1 (SMD = 1.17, 95% CI 0.73–1.61, *p* < 0.001; *I*^2^ = 95.8%, *p* < 0.001), PECAM-1 (SMD = 0.82, 95% CI 0.57–1.08, *p* < 0.001; *I*^2^ = 0.0%, *p* = 0.90), E-selectin (SMD = 0.64, 95% CI 0.42–0.86, *p* < 0.001; *I*^2^ = 75.0%, *p* < 0.001), and P-selectin (SMD = 1.06, 95% CI 0.50–1.60, *p* < 0.001; *I*^2^ = 84.8%, *p* < 0.001), but not L-selectin. In meta-regression and subgroup analysis, significant associations were observed between the effect size and use of glucocorticoids (ICAM-1), erythrocyte sedimentation rate (VCAM-1), study continent (VCAM-1, E-selectin, and P-selectin), and matrix assessed (P-selectin).

**Conclusions:**

The results of our study support a significant role of cell adhesion molecules in mediating the interplay between RA and atherosclerosis. Further studies are warranted to determine whether the routine use of these biomarkers can facilitate the detection and management of early atherosclerosis in this patient group.

*PROSPERO Registration Number*: CRD42023466662.

**Supplementary Information:**

The online version contains supplementary material available at 10.1007/s00011-023-01837-6.

## Introduction

Despite significant advances, particularly over the last two decades, in diagnosis, treatment, and follow-up, patients with rheumatoid arthritis (RA) continue to experience poor quality of life and inadequate social engagement [1–5]. There is also increasing evidence that the coexistence of RA and specific comorbidities exerts an additional public health and financial burden on patients and the healthcare workforce [6–8]. In particular, the link between RA and atherosclerosis is well established given the high prevalence of cardiovascular risk factors [9–14], the high incidence of myocardial infarction and stroke [15–19], and the recognition that cardiovascular disease represents the leading cause of mortality in RA patients [20, 21]. To further corroborate the link between RA and atherosclerosis, a considerable body of research has demonstrated significant alterations in the endothelial synthesis of the critical endogenous messenger, nitric oxide [22, 23], impaired endothelial and flow-mediated vasodilatation [24–27], intima–media thickening [28], arterial stiffening [29], and increased risk of hypertension in RA patients [30]. These abnormalities are often observed in the early phases of atherosclerosis, reflecting a state of endothelial activation and early damage of the tunica intima of the arterial wall [31, 32], and therefore their detection might instigate timely prevention strategies [33–35].

At a cellular and molecular level, the early stages of atherosclerosis are characterized by the adhesion of leukocytes and lymphocytes to the endothelium. This, in turn, facilitates the migration of these cells to the tunica intima [36, 37]. The process of cellular adhesion to the endothelium is mediated by a number of molecules, including the immunoglobulin-like vascular cell adhesion molecule-1 (VCAM-1), the intercellular vascular adhesion molecule-1 (ICAM-1), and the platelet endothelial cell adhesion molecule-1 (PECAM-1) [38–41]. VCAM-1 is primarily expressed in endothelial cells and macrophages and its binding to integrin α_4_β_1_ mediates its biological effects [42, 43]. ICAM-1 is upregulated in the presence of excess inflammation and binds to the leukocyte specific β_2_ integrins [44, 45]. PECAM-1 is expressed in leukocytes, platelets, and endothelial cells, and favors the migration of leukocytes through the translocation of the integrin α_6_β_1_ [46]. The immunoglobulin-like vascular cell adhesion molecules can be measured in plasma or serum [41, 47, 48], and their concentrations have been shown to be positively associated with cardiovascular risk [49–53]. Another group of molecules facilitating cell adhesion to the endothelium includes the selectins, specifically P-selectin, expressed in platelets and endothelial cells, L-selectin, expressed in leukocytes, and E-selectin, expressed in endothelial cells [54–56]. Selectins mediate the rolling of monocytes, neutrophils, and lymphocytes [57, 58], can also be measured in plasma or serum, and have been shown to play an important pathophysiological role in RA, angiogenesis, and atherosclerosis [59–68].

Therefore, given the robust association between RA and atherosclerosis and the potential utility of cell adhesion molecules in the identification of early, subclinical atherosclerosis, we conducted a systematic review and meta-analysis of the circulating concentrations of VCAM-1, ICAM-1, PECAM-1, P-selectin, L-selectin, and E-selectin in RA patients and healthy controls. We hypothesized that RA is associated with a significant upregulation of cell adhesion molecules, suggesting endothelial activation and dysfunction in this patient group.

## Methods

### Search strategy and study selection

We searched PubMed, Scopus, and Web of Science from inception to 31 July 2023 for relevant articles using the following terms and their combinations: “rheumatoid arthritis” AND “soluble cell adhesion molecules” OR “intercellular adhesion molecule-1” OR “ICAM-1” OR “sICAM-1” OR “ICAM” OR “vascular cell adhesion molecule-1” OR “VCAM-1” OR “sVCAM-1” OR “VCAM” OR “platelet endothelial cell adhesion molecule-1” OR “PECAM-1” OR “sPECAM-1” OR “PECAM” OR “selectin” or “P-selectin” OR “sP-selectin” OR “L-selectin” OR “sL-selectin” OR “E-selectin” OR “sE-selectin”. Two investigators independently screened the abstracts and, if relevant, the full articles, including their references, according to the following criteria: (a) assessment of soluble ICAM-1, VCAM-1, PECAM-1, E-selectin, L-selectin, and P-selectin in plasma or serum, (b) comparison of RA patients and healthy controls in a case–control study, (c) age of participants ≥ 18 years, (d) publications in English language, and (e) full-text article available.

The following information was independently extracted from selected manuscripts: year of publication, first author, study country and continent, number of RA patients and controls, age, sex distribution, C-reactive protein (CRP), erythrocyte sedimentation rate (ESR), RA duration, disease activity score-28 (DAS-28), matrix used for assessment (serum or plasma), method used to measure adhesion molecules, and use of methotrexate, glucocorticoids, and disease-modifying antirheumatic drugs (DMARDs).

The risk of bias was assessed using the Joanna Briggs Institute Critical Appraisal Checklist for analytical studies and the certainty of evidence using the Grades of Recommendation, Assessment, Development, and Evaluation (GRADE) Working Group system [69, 70]. We complied with the Preferred Reporting Items for Systematic Reviews and Meta-Analyses (PRISMA) 2020 statement (Supplementary Tables S1 and S2) [71]. The protocol was registered in the International Prospective Register of Systematic Reviews (PROSPERO Registration No. CRD42023466662).

### Statistical analysis

We calculated standardized mean differences (SMDs) and 95% confidence intervals (CIs) to generate forest plots of continuous data and investigate differences in the concentrations of cell adhesion molecules between RA patients and healthy controls (a *p* value < 0.05 was considered statistically significant) [72–74]. The heterogeneity of SMD across studies was evaluated using the Q-statistic (a *p* value *p* < 0.10 was considered statistically significant). Heterogeneity was considered low when *I*^2^ ≤ 25%, moderate when 25% < *I*^2^ < 75%, and high when *I*^2^ ≥ 75%. A random-effects model based on the inverse-variance method was used in case of high heterogeneity [75, 76]. The stability of the results was assessed using sensitivity analysis [77]. The Begg’s and Egger’s tests (a *p*-value < 0.05 was considered statistically significant) and the “trim-and-fill” method were used to assess publication bias [78–80]. Univariate meta-regression and subgroup analyses were performed to investigate associations between the effect size and the following parameters: year of publication, geographical area where the study was conducted, sample size, age, sex distribution, CRP, ESR, RA duration, DAS-28, sample matrix (serum or plasma), analytical method, and the use of methotrexate, glucocorticoids, and DMARDs. All statistical analyses were performed using Stata 14 (Stata Corp., College Station, TX, USA).

## Results

### Study selection

**Fig. 1:**
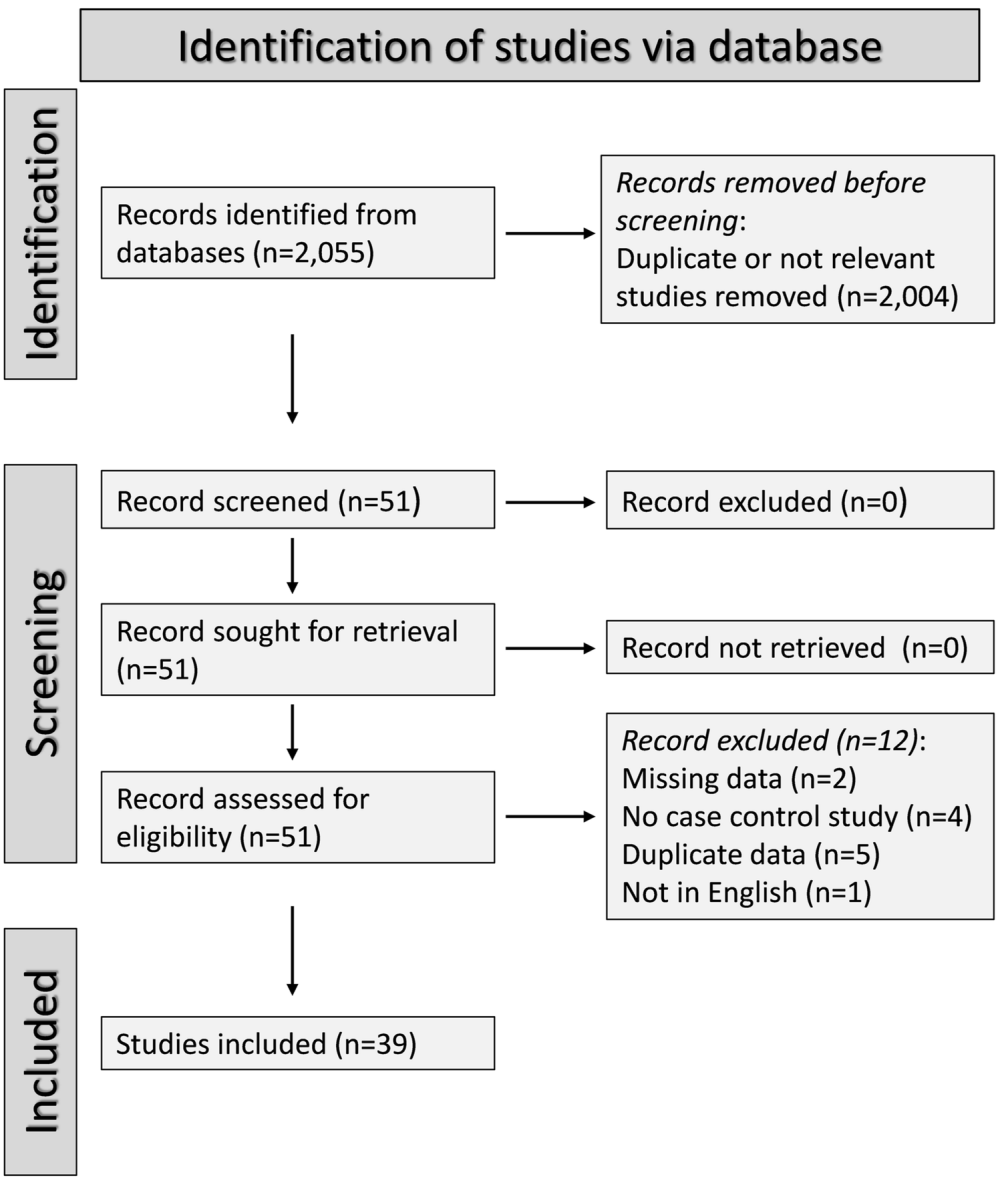
PRISMA 2020 flow diagram

Our search identified 2055 articles. Of them, 2004 were excluded following the initial screening as they were either duplicates or not relevant. A full-text review of the remaining 51 articles led to the further exclusion of 5 studies because of duplicate data, 4 because they were not case–control, 2 because they had missing data, and 1 because it was not written in English. Therefore, 39 studies were selected for analysis (Fig. 1 and Table 1) [72, 81–118]. The initial level of certainty was rated as low (rating 2) given the cross-sectional design of all studies.

**Table 1:** Study characteristics

Study	Healthy controls					Patients with rheumatoid arthritis				
	*n*	Age (years)	M/F	ICAM-1	E-selectin	*n*	Age (years)	M/F	ICAM	E-selectin
				VCAM-1	L-selectin				VCAM	L-selectin
				PECAM-1 (Mean ± SD)	P-selectin (Mean ± SD)				PECAM-1 (Mean ± SD)	P-selectin (Mean ± SD)
Cush et al. [81]	7	NR	NR	191 ± 39	NR	61	50	11/50	548 ± 434	NR
				NR	NR				NR	NR
				NR	NR				NR	NR
Machold et al. [82]	82	48	37/45	313 ± 78	NR	46	53	6/34	326 ± 98	NR
				NR	NR				NR	NR
				NR	NR				NR	NR
Blann et al. [83]	80	45	39/41	319 ± 130	57.5 ± 24	40	50	9/31	476 ± 227	76.3 ± 32
				746 ± 330	NR				497 ± 182	NR
				NR	NR				NR	NR
Vosckuyl et al. [84]	100	NR	NR	403 ± 103	53 ± 16	47	58	11/36	418 ± 76	91 ± 50
				NR	NR				NR	NR
				NR	NR				NR	NR
Kuryliszyn-Moskal et al. [85]	20	48	4/16	258 ± 43	NR	79	52	16/63	405 ± 114	NR
				NR	NR				NR	NR
				NR	NR				NR	NR
Littler et al. [86]	10	36	NR	184 ± 25	28.8 ± 9.6	22	57	NR	309 ± 100	42.9 ± 19.2
				524 ± 156	980 ± 251				828 ± 294	997 ± 352
				NR	104 ± 55				NR	199 ± 87
Ertenli et al. [87]	24	39	4/20	NR	NR	20	38	3/17	NR	NR
				NR	NR				NR	NR
				NR	176 ± 40				NR	464 ± 144
Veale et al. [88]	13	63	1/12	238 ± 94	48.2 ± 14.0	13	63	1/12	345 ± 107	49.3 ± 17.0
				493 ± 412	NR				816 ± 153	NR
				NR	114 ± 37				NR	333 ± 174
Salih et al. [89]	25	NR	NR	578 ± 204	32 ± 10	29	62	9/20	869 ± 214	45 ± 27
				NR	NR				NR	NR
				NR	NR				NR	NR
Sfikakis et al. [90]	40	Matched	Matched	NR	NR	25	49	5/20	NR	NR
				NR	930 ± 251				NR	997 ± 352
				NR	245 ± 97				NR	370 ± 160
Jonsson et al. [91]	39	Matched	Matched	247 ± 50	53.7 ± 16.9	39	52	9/30	364 ± 156	63.1 ± 24.9
				NR	NR				NR	NR
				NR	NR				NR	NR
Cogalgil et al. [92]	30	43	10/20	232 ± 25	NR	42	46	8/34	497 ± 65	NR
				NR	NR				NR	NR
				NR	NR				NR	NR
El Miedany et al. [93]	25	Matched	Matched	NR	31.4 ± 7.6	40	53	8/32	NR	45.8 ± 12.9
				586 ± 87	NR				816 ± 125	NR
				NR	NR				NR	NR
Witkowska et al. [94]	80	44	0/80	267 ± 45	NR	37	49	0/44	353 ± 131	NR
				NR	NR				NR	NR
				NR	NR				NR	NR
Ates et al. [95]	16	50	4/12	NR	24.9 ± 12.9	34	53	10/24	NR	43.7 ± 30.9
				NR	672 ± 140				NR	378 ± 168
				NR	292 ± 199				NR	383 ± 198
Sharaki et al. [96]	15	Matched	Matched	NR	28 ± 9	30	46	4/26	NR	67 ± 29
				NR	NR				NR	NR
				NR	NR				NR	NR
Dessein et al. [97]	80	44	16/64	350 ± 194	NR	74	57	10/64	465 ± 215	NR
				583 ± 204	NR				991 ± 422	NR
				NR	NR				NR	NR
Macias et al. [98]	20	Matched	Matched	NR	NR	36	53	8/28	NR	NR
				562 ± 186	NR				1239 ± 341	NR
				NR	NR				NR	NR
Navarro-Hernandez et al. [99]	60	NR	NR	NR	39 ± 17.8	60	42	5/55	NR	91.7 ± 71.0
				NR	NR				NR	NR
				NR	NR				NR	NR
Pahor et al. [100]	40	42	0/40	253 ± 33	NR	70	42	0/70	298 ± 102	NR
				200 ± 61	NR				646 ± 151	NR
				NR	NR				NR	NR
Kao et al. [101]	105	52	0/105	248 ± 42	NR	105	52	0/105	280 ± 79	NR
				NR	NR				NR	NR
				NR	NR				NR	NR
Pamuk et al. [102]	19	49	6/13	NR	32.1 ± 14.6	27	52	6/21	NR	34.2 ± 12.5
				NR	NR				NR	NR
				NR	NR				NR	NR
Wållberg-Jonsson et al. [103]	30	Matched	Matched	570 ± 207	49.2 ± 13.0	30	54	7/23	593 ± 312	69.8 ± 32.0
				677 ± 162	1090 ± 171				773 ± 241	1074 ± 239
				26.7 ± 3.0	103 ± 32				29.6 ± 4.2	105 ± 30
Foster et al. [104]	45	54	14/31	181 ± 52	50 ± 16	57	58	21/36	222 ± 54	66 ± 32
				371 ± 151	NR				448 ± 148	NR
				NR	NR				NR	NR
Navarro-Hernandez et al. [105]	60	39	9/51	151 ± 176	NR	60	46	9/51	496 ± 773	NR
				302 ± 227	NR				569 ± 707	NR
				NR	NR				NR	NR
Pemberton et al. [106]	48	56	0/48	NR	82.7 ± 39.3	46	57	0/46	NR	104.5 ± 19.7
				234 ± 45	NR				259 ± 54	NR
				NR	18.5 ± 6.8				NR	20.7 ± 6.6
Rho et al. [107]	92	53	34/58	127 ± 47	15.3 ± 8.1	169	54	52/117	165 ± 57	20.8 ± 8.0
				939 ± 261	NR				953 ± 336	NR
				NR	NR				NR	NR
Olewicz-Gawlik et al. [108]	30	NR	2/28	NR	30.4 ± 17.9	40	60	4/36	NR	27.4 ± 11.6
				NR	583 ± 149				NR	1967 ± 915
				NR	39.1 ± 18.4				NR	549 ± 510
Södergren et al. [109]	44	46	10/34	291 ± 67	53.8 ± 16.7	79	48	15/64	354 ± 132	51.8 ± 19.2
				631 ± 173	1280 ± 273				743 ± 238	1252 ± 306
				NR	NR				NR	NR
de Groot et al. [110]	49	59	19/30	NR	NR	49	58	19/30	NR	NR
				406 ± 117	NR				541/159	NR
				NR	NR				NR	NR
Santos et al. [111]	124	47	0/124	454 ± 233	NR	107	50	0/107	654 ± 336	NR
				1068 ± 383	NR				1134 ± 435	NR
					NR					NR
Klimek et al. [112]	29	32	16/13	NR	12.45 ± 8.02	29	41	7/22	NR	17.65 ± 8.67
				613 ± 148	NR				744 ± 190	NR
					NR					NR
Pamuk et al. [113]	94	40	16/78	NR	NR	100	55	19/81	NR	NR
				NR	NR				NR	NR
				8.72 ± 2.07	NR				11.18 ± 3.6	NR
Wang et al. [72]	30	44	15/15	NR	NR	120	43	62/58	NR	NR
				421 ± 74	NR				1126 ± 126	NR
				NR	NR				NR	NR
Rodriguez-Carrio et al. [114]	175	51	35/140	159 ± 104	NR	212	54	37/175	230 ± 141	NR
				NR	NR				NR	NR
				NR	NR				NR	NR
Sarithala et al. [115]	70	Matched	Matched	499 ± 169	NR	134	50	30/104	1314 ± 1090	NR
				NR	NR				NR	NR
				NR	NR				NR	NR
Bezuidenhout et al. [116]	25	53	8/17	306 ± 97	NR	30	53	6/24	384 ± 118	NR
				341 ± 104	NR				348 ± 95	NR
				NR	NR				NR	NR
Salem et al. [117]	50	39	6/44	NR	NR	50	40	5/45	NR	NR
				760 ± 169	NR				1022 ± 200	NR
				NR	NR				NR	NR
Salem et al. [117]	50	39	6/44	NR	NR	50	36	4/46	NR	NR
				760 ± 169	NR				1494 ± 385	NR
				NR	NR				NR	NR
Gerasimova et al. [118]	100	Matched	Matched	292 ± 72	NR	275	51	32/243	337 ± 97	NR
				901 ± 520	NR				1460 ± 719	NR
				NR	NR				NR	NR

NR, not reported; M/F, male to female ratio; VCAM-1, vascular cell adhesion molecule-1; ICAM-1, intercellular adhesion molecule-1; PECAM-1, platelet endothelial cell adhesion molecule-1

#### ICAM-1

Twenty-four studies investigated ICAM-1 in a total of 1857 RA patients (mean age 52 years, 84% females) and 1476 healthy controls (mean age 48 years, 81% females) [81–86, 88, 89, 91, 92, 94, 97, 100, 101, 103–105, 107, 109, 111, 114–116, 118]. Eighteen studies were conducted in Europe [82–86, 88, 89, 91, 94, 100, 103, 104, 109, 111, 114–116, 118], 4 in America [81, 101, 105, 107], 1 in Asia [92], and 1 in Africa [97]. ICAM-1 was measured using an enzyme-linked immunosorbent assay (ELISA) in all but one study, which did not provide relevant details regarding the analytical method used [100]. Eighteen studies measured ICAM-1 in serum [81–86, 88, 89, 91, 92, 94, 105, 107, 109, 111, 114, 116, 118], 2 in plasma [104, 115], while the remaining 4 did not provide relevant information [97, 101, 103, 113]. RA duration was reported in 18 studies and ranged between 0.25 and 23 years [81, 82, 84, 85, 88, 89, 92, 94, 97, 100, 101, 103, 105, 109, 111, 114, 115, 118], whereas DAS-28, reported in six studies, ranged between 2.8 and 6.23 [103, 105, 109, 114, 116, 118]. Eleven studies reported data on methotrexate treatment [85, 86, 88, 91, 92, 94, 104, 111, 114, 116, 118], 18 on glucocorticoid treatment [81, 84, 86, 88, 91, 92, 94, 97, 100, 101, 103–105, 109, 114, 116, 118], and 14 on DMARDs treatment [81, 84, 86, 88, 89, 91, 94, 97, 100, 103, 105, 109, 116, 118]. The risk of bias was low in 11 studies [89, 97, 100, 101, 104, 105, 107, 109, 111, 114, 116], moderate in 12 [81–86, 88, 91, 92, 94, 115, 118], and high in the remaining 1 [103] (Table 2).

**Table 2:** Assessment of the risk of bias

Study	Were the criteria for inclusion clearly defined?	Were the subjects and the setting described in detail?	Was the exposure measured in a valid and reliable way?	Were objective and standard criteria used for measurement of the condition?	Were confounding factors identified?	Were strategies to deal with confounding factors stated?	Were the outcomes measured in a valid and reliable way?	Was appropriate statistical analysis used?	Risk of bias
Cush et al. [81]	No	No	Yes	Yes	No	No	Yes	Yes	Moderate
Machold et al. [82]	No	No	Yes	Yes	No	No	Yes	Yes	Moderate
Blann et al. [83]	Yes	No	Yes	Yes	No	No	Yes	Yes	Moderate
Vosckuyl et al. [84]	No	No	Yes	Yes	No	No	Yes	Yes	Moderate
Kuryliszyn-Moskal et al. [85]	Yes	No	Yes	Yes	No	No	Yes	Yes	Moderate
Littler et al. [86]	No	No	Yes	Yes	No	No	Yes	Yes	Moderate
Ertenli et al. [87]	No	No	Yes	Yes	No	No	Yes	Yes	Moderate
Veale et al. [88]	No	No	Yes	Yes	No	No	Yes	Yes	Moderate
Salih et al. [89]	Yes	Yes	Yes	Yes	No	No	Yes	Yes	Low
Sfikakis et al. [90]	No	No	Yes	Yes	No	No	Yes	Yes	Moderate
Jonsson et al. [91]	No	No	Yes	Yes	No	No	Yes	Yes	Moderate
Cogalgil et al. [92]	No	No	Yes	Yes	No	No	Yes	Yes	Moderate
El Miedany et al. [93]	Yes	Yes	Yes	Yes	No	No	Yes	Yes	Low
Witkowska et al. [94]	No	No	Yes	Yes	No	No	Yes	Yes	Moderate
Ates et al. [95]	No	No	Yes	Yes	No	No	Yes	Yes	Moderate
Sharaki et al. [96]	No	No	Yes	Yes	No	No	Yes	Yes	Moderate
Dessein et al. [97]	No	No	Yes	Yes	Yes	Yes	Yes	Yes	Low
Macias et al. [98]	Yes	Yes	Yes	Yes	No	No	Yes	Yes	Low
Navarro-Hernandez et al. [99]	No	No	Yes	Yes	No	No	Yes	Yes	Moderate
Pahor et al. [100]	No	No	Yes	Yes	Yes	Yes	Yes	Yes	Low
Kao et al. [101]	No	No	Yes	Yes	Yes	Yes	Yes	Yes	Low
Pamuk et al. 2008, Turkey [102]	No	No	Yes	Yes	No	No	Yes	Yes	Moderate
Wållberg-Jonsson et al. [103]	No	No	Yes	No	No	No	Yes	Yes	High
Foster et al. [104]	Yes	Yes	Yes	Yes	No	No	Yes	Yes	Low
Navarro-Hernandez et al. [105]	Yes	Yes	Yes	Yes	No	No	Yes	Yes	Low
Pemberton et al. [106]	No	No	Yes	No	No	No	Yes	Yes	High
Rho et al. [107]	No	No	Yes	Yes	Yes	Yes	Yes	Yes	Low
Olewicz-Gawlik et al. [108]	No	No	Yes	No	No	No	Yes	Yes	High
Södergren et al. [109]	No	No	Yes	Yes	Yes	Yes	Yes	Yes	Low
de Groot et al. [110]	Yes	Yes	Yes	Yes	Yes	Yes	Yes	Yes	Low
Santos et al. [111]	Yes	Yes	Yes	Yes	Yes	Yes	Yes	Yes	Low
Klimek et al. [112]	Yes	Yes	Yes	Yes	Yes	Yes	Yes	Yes	Low
Pamuk et al. [113]	Yes	Yes	Yes	Yes	Yes	Yes	Yes	Yes	Low
Wang et al. [72]	No	No	Yes	No	No	No	Yes	Yes	High
Rodriguez-Carrio et al. [114]	Yes	Yes	Yes	Yes	Yes	Yes	Yes	Yes	Low
Sarithala et al. [115]	No	Yes	Yes	Yes	No	No	Yes	Yes	Moderate
Bezuidenhout et al. [116]	Yes	Yes	Yes	Yes	Yes	Yes	Yes	Yes	Low
Salem et al. [117]	Yes	Yes	Yes	Yes	No	No	Yes	Yes	Low
Gerasimova et al. [118]	No	Yes	Yes	Yes	No	No	Yes	Yes	Moderate

**Fig. 2:**
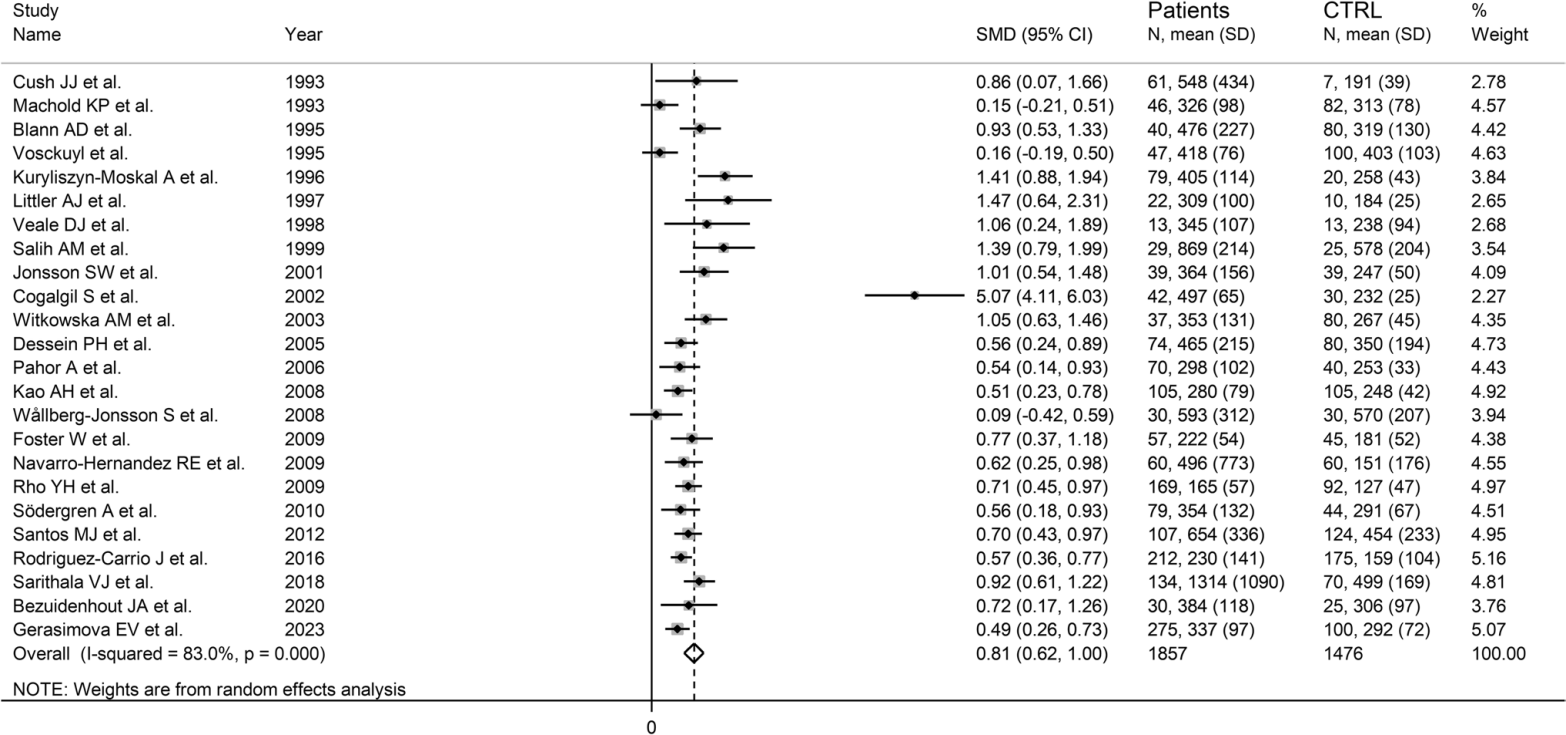
Forest plot of studies investigating ICAM-1 in RA patients and healthy controls

The forest plot showed that RA patients had significantly higher ICAM-1 concentrations when compared to healthy controls (SMD = 0.81, 95% CI 0.62–1.00, *p* < 0.001; *I*^2^ = 83.0%, *p* < 0.001; Fig. 2). The results were stable in sensitivity analysis, with pooled SMD values ranging between 0.68 and 0.84 (Supplementary Fig. S1). There was significant publication bias according to the Begg’s test (*p* = 0.003) and the Egger’s test (*p* = 0.007). The “trim-and-fill” method identified six missing studies to be added to the left side of the funnel plot to ensure symmetry (Supplementary Fig. S2). The resulting effect size remained significant (SMD = 0.60, 95% CI 0.38–0.81, *p* < 0.001).

**Fig. 3:**
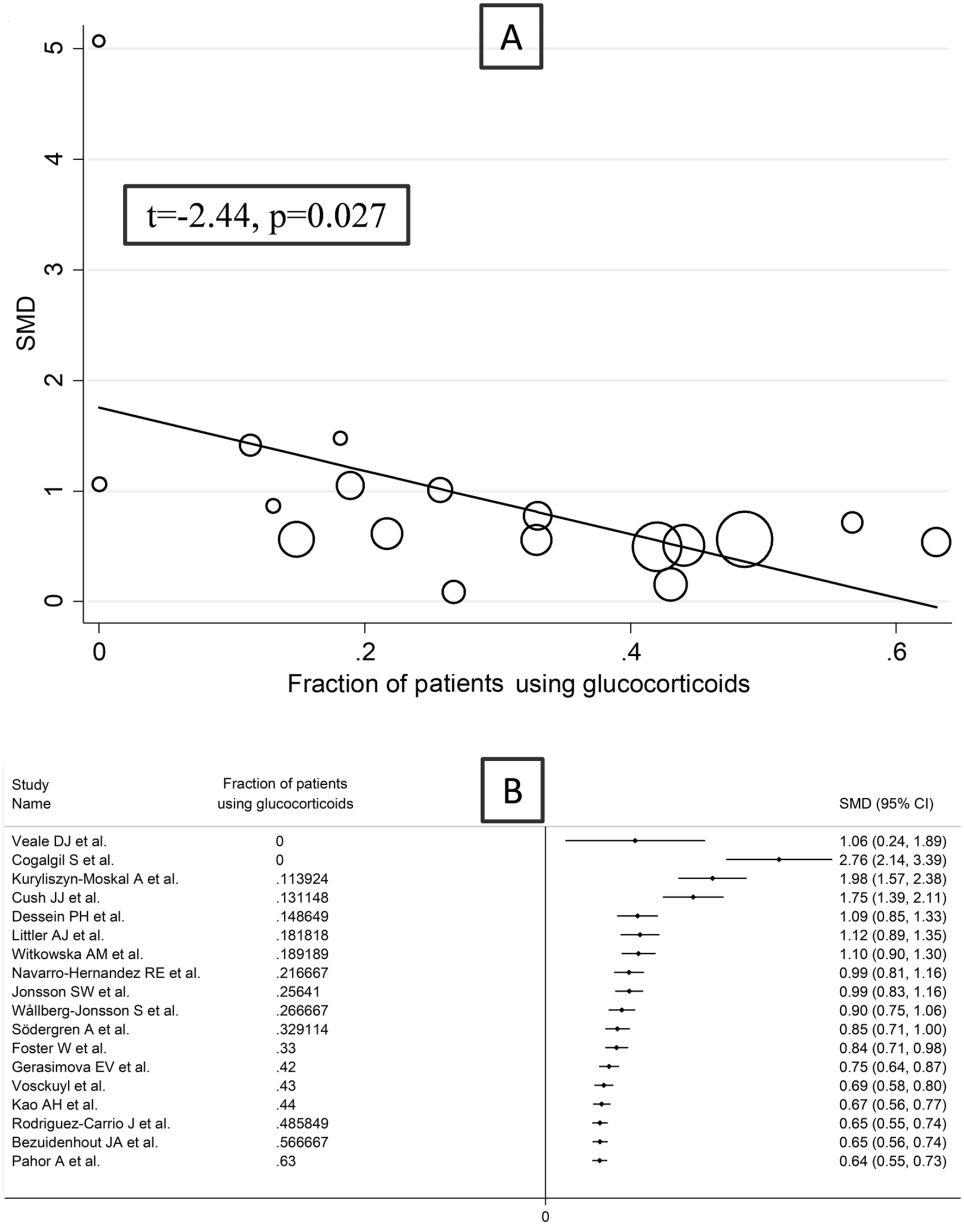
Bubble plot reporting univariate meta-regression analysis between the effect size and the proportion of patients using glucocorticoids (**A**) and cumulative meta-analysis of ICAM-1 concentrations based on the proportion of patients using glucocorticoids (**B**)

In meta-regression, there were non-significant associations between the effect size and age (*t* = 0.50, *p* = 0.62), sex distribution (*t* = − 0.81, *p* = 0.43), publication year (*t* = − 0.81, *p* = 0.43), number of participants (*t* = − 1.29, *p* = 0.21), CRP (*t* = 1.41, *p* = 0.19), ESR (*t* = 1.08, *p* = 0.31), RA duration (*t* = − 1.20, *p* = 0.25), or use of methotrexate (*t* = − 0.96, *p* = 0.36), and DMARDs (*t* = − 0.49, *p* = 0.63). By contrast, there was a significant inverse association between the effect size and the use of glucocorticoids (*t* = − 2.44, *p* = 0.027) (Fig. 3a, b). In subgroup analysis, there were non-significant differences (*p* = 0.70) in pooled SMD between European (SMD = 0.72, 95% CI 0.56–0.88, *p* < 0.001; *I*^2^ = 67.6%, *p* < 0.001) and American studies (SMD = 0.62, 95% CI 0.46–0.79, *p* < 0.001; *I*^2^ = 0.0%, *p* = 0.69; Supplementary Fig. S3) with a virtually absent heterogeneity in the American subgroup. Similarly, there were non-signficant differences (*p* = 0.83) in pooled SMD between studies measuring serum (SMD = 0.93, 95% CI 0.67–1.18, *p* < 0.001; *I*^2^ = 86.4%, *p* < 0.001) and plasma (SMD = 0.86, 95% CI 0.62–1.11, *p* < 0.001; *I*^2^ = 0.0%, *p* = 0.58; Supplementary Fig. S4), with a virtually absent heterogeneity in the plasma subgroup.

The overall level of certainty was upgraded to moderate (rating 3) after considering the low-moderate risk of bias in most studies (no change), the high but partly explainable heterogeneity (no change), the lack of indirectness (no change), the relatively large effect size (SMD = 0.81, upgrade one level) [119], and the presence of publication bias which was addressed with the “trim-and-fill” method (no change).

#### VCAM-1

Twenty studies (21 comparator groups) investigated VCAM-1 in a total of 1446 RA patients (mean age 50 years, 81% females) and 1044 healthy controls (mean age 48 years, 79% females) [72, 83, 86, 88, 93, 97, 98, 100, 103–107, 109–112, 116–118]. Fourteen studies were conducted in Europe [83, 86, 88, 98, 100, 103, 104, 106, 109–112, 116, 118], 3 in Africa [93, 97, 117], 2 in America [105, 107], and 1 in Asia [72]. VCAM-1 was measured using an ELISA assay in all but one study for all studies except for one in which authors did not declare the method employed [100]. Fourteen studies investigated ICAM-1 in serum [72, 83, 86, 88, 93, 98, 105, 107, 109–111, 116–118] and 2 in plasma [104, 112]. No relevant information regarding the matrix analysed was provided in the remaining four studies [97, 100, 103, 106]. RA duration was reported in 12 studies and ranged between 0.25 and 23 years [88, 93, 97, 98, 100, 103, 105, 109–112, 118], whereas the DAS-28 was reported in 11 study comparators and ranged between 2.3 and 6.23 [98, 103, 105, 106, 109, 110, 112, 116–118]. The use of methotrexate was reported in 10 studies [72, 86, 88, 98, 104, 110, 111, 116–118], glucocorticoids in 14 [72, 86, 88, 97, 100, 103–106, 109, 112, 116–118], and DMARDs in 13 [72, 86, 88, 93, 97, 100, 103, 105, 106, 109, 110, 116, 117]. The risk of bias was low in 13 studies [93, 97, 98, 100, 104, 105, 107, 109–112, 116, 117], moderate in 4 [83, 86, 88, 118], and high in the remaining 3 [72, 103, 106] (Table 2).

**Fig. 4:**
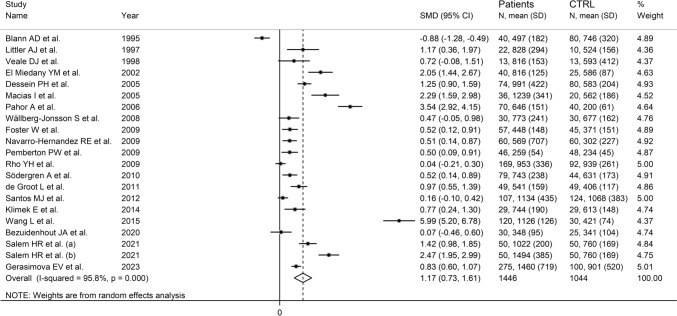
Forest plot of studies investigating VCAM-1 investigating ICAM-1 in RA patients and controls

The forest plot showed that VCAM-1 concentrations were significantly higher in RA patients when compared to controls (SMD = 1.17, 95% CI 0.73–1.61, *p* < 0.001; *I*^2^ = 95.8%, *p* < 0.001; Fig. 4). The pooled SMD values were stable in sensitivity analysis, ranging between 0.94 and 1.27 (Supplementary Fig. S5). There was a significant publication bias (Begg’s test, *p* = 0.006, and Egger’s test, *p* = 0.009). The “trim-and-fill” method identified eight missing studies to be added to the left side of the funnel plot to ensure symmetry (Supplementary Fig. S6). However, the resulting effect size was no longer significant (SMD = 0.39, 95% CI − 0.11 to 0.88, *p* = 0.12).

**Fig. 5:**
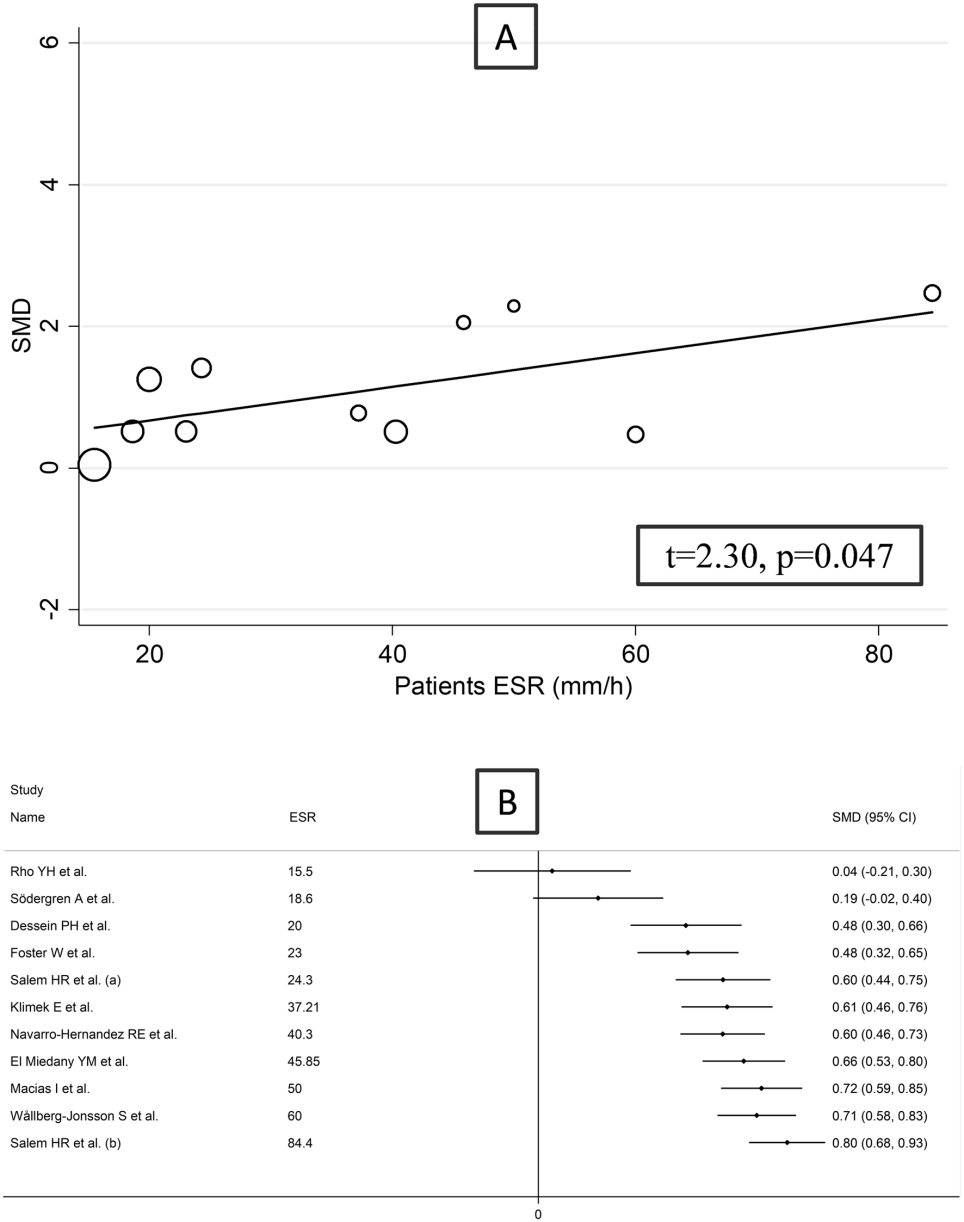
Bubble plot reporting univariate meta-regression analysis between the effect size and ESR values of RA patients (**A**) and cumulative meta-analysis of VCAM-1 concentrations based on ESR values (**B**)

**Fig. 6:**
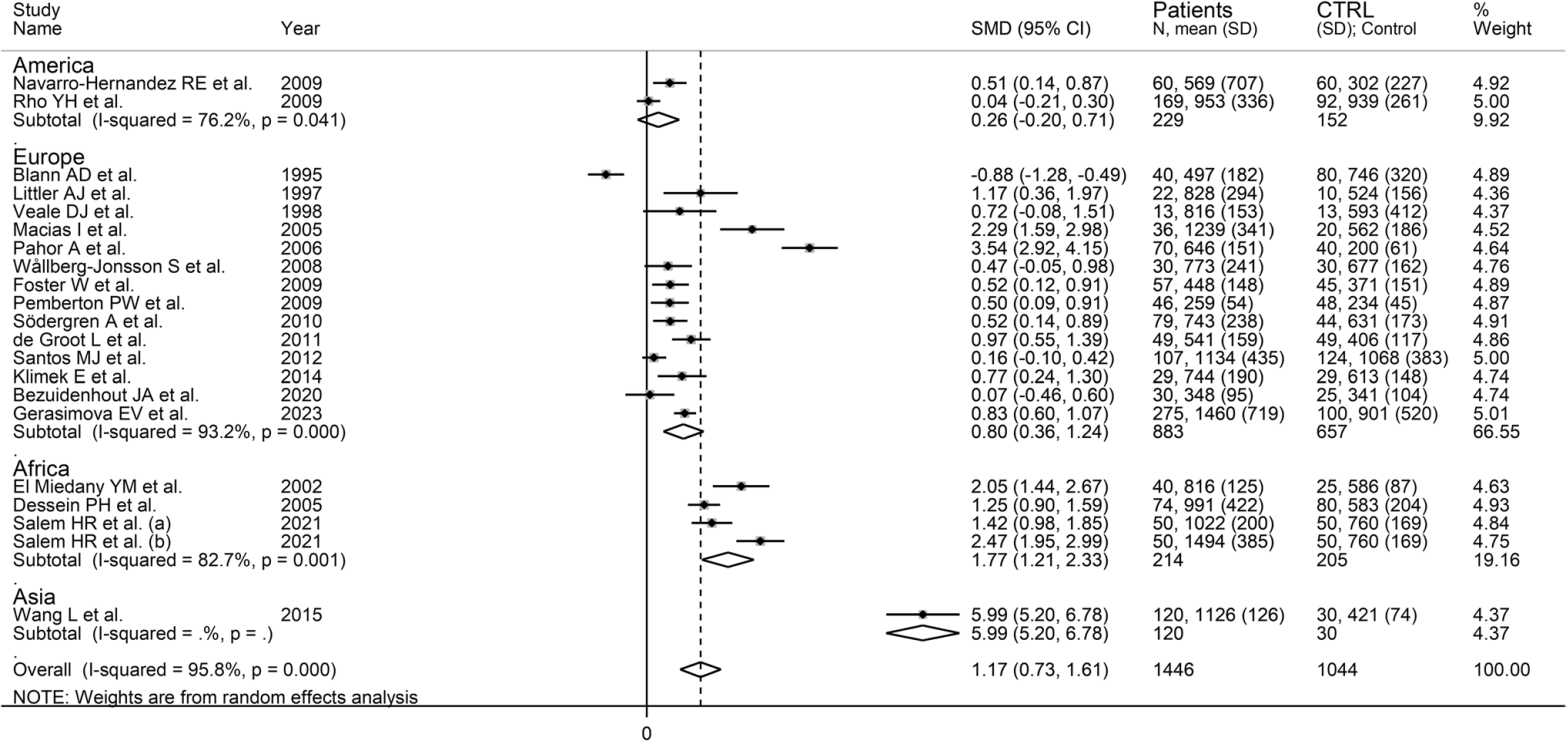
Forest plot of studies investigating VCAM-1 in RA patients and controls according to study continent

Non-significant associations were observed in the meta-regression between the effect size and age (*t* = − 0.86, *p* = 0.40), sex distribution (*t* = 1.38, *p* = 0.19), year of publication (*t* = 0.78, *p* = 0.45), sample size (*t* = − 0.33, *p* = 0.74), CRP (*t* = 1.03, *p* = 0.33), RA duration (*t* = − 0.10, *p* = 0.92), or use of methotrexate (*t* = − 1.62, *p* = 0.14) and glucocorticoids (*t* = − 1.05, *p* = 0.32). However, there was a trend toward a significant association between the effect size and DMARDs use (*t* = − 2.00, *p* = 0.07) and a significant association between SMD and ESR (*t* = 2.30, *p* = 0.047; Fig. 5a, b). In subgroup analysis, there was a significant difference (*p* < 0.001) in pooled SMD between American (SMD = 0.26, 95% CI − 0.20 to 0.71, *p* = 0.27; *I*^2^ = 76.2%, *p* = 0.041), European (SMD = 0.80, 95% CI 0.36–1.24, *p* < 0.001; *I*^2^ = 93.2%, *p* < 0.001), and African studies (SMD = 1.77, 95% CI 1.21–2.33, *p* < 0.001; *I*^2^ = 82.7%, *p* < 0.001; Fig. 6). By contrast, there were non-significant differences (*p* = 0.63) in pooled SMD between studies investigating serum (SMD = 1.18, 95% CI 0.63–1.73, *p* < 0.001; *I*^2^ = 96.3%, %, *p* < 0.001) and plasma (SMD = 0.61, 95% CI 0.29–0.92, *p* = 0.22; *I*^2^ = 0.0%, *p* = 0.455; Supplementary Fig. S7), with a virtual absence of heterogeneity in the plasma subgroup.

The overall level of certainty remained low (rating 2) after considering the low–moderate risk of bias in most studies (no change), the high but partly explainable heterogeneity (no change), the lack of indirectness (no change), the relatively large effect size (SMD = 1.17, upgrade one level) [119], and the presence of publication bias which was not fully addressed with the “trim-and-fill” method (downgrade one level).

#### PECAM-1

**Fig. 7:**
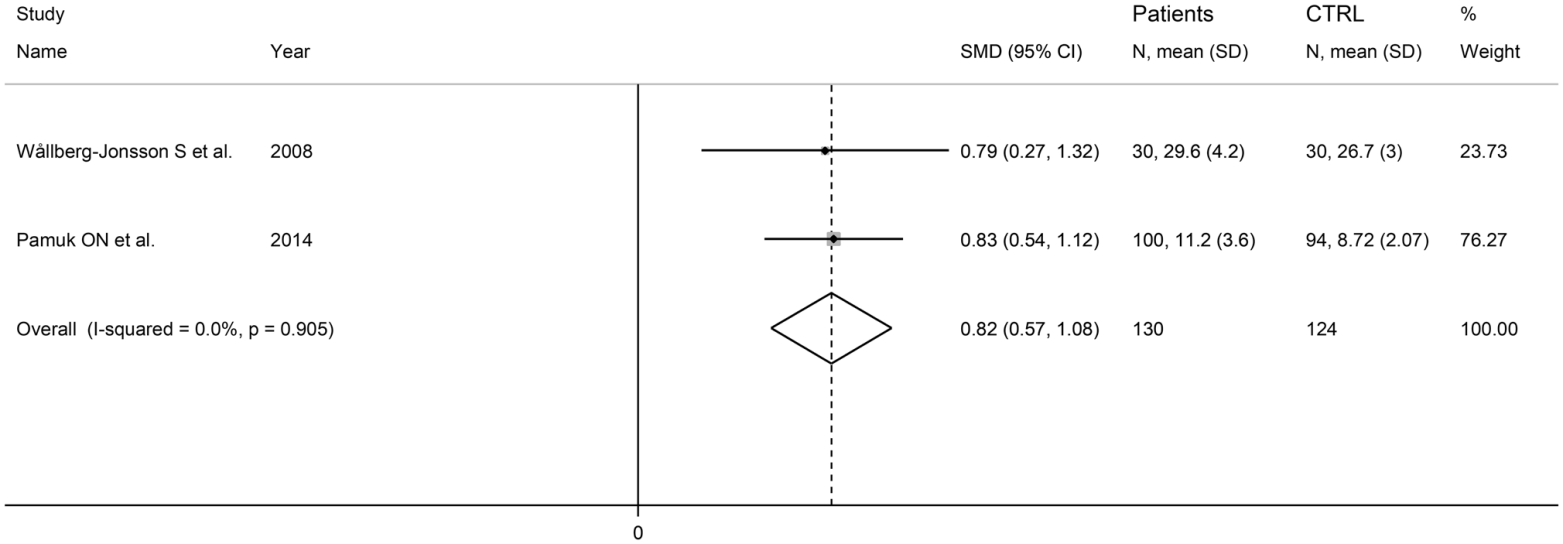
Forest plot of studies investigating PECAM-1 in RA patients and controls

Two studies, one conducted in Europe [103], and the other in Asia [113], investigated PECAM-1 in a total of 130 RA patients (mean age 55 years, 80% females) and 124 healthy controls (mean age 43 years, 81% females). PECAM-1 was measured using an ELISA in both studies, in serum in one study [113], whereas the second study did not provide any details regarding the matrix assessed [103]. The risk of bias was low in one study [113] and high in the other [103] (Table 2). The forest plot showed that RA patients had significantly higher PECAM-1 concentrations when compared to controls (SMD = 0.82, 95% CI 0.57–1.08, *p* < 0.001; *I*^2^ = 0.0%, *p* = 0.905; Fig. 7). Sensitivity analysis, assessment of publication bias, and meta-regression and subgroup analysis could not be conducted because of the limited number of studies.

The overall level of certainty remained low (rating 2) after considering the low risk of bias in one of the two studies (no change), the virtually absent heterogeneity (no change), the lack of indirectness (no change), the relatively large effect size (SMD = 0.82, upgrade one level) [119], and the lack of assessment of publication bias (downgrade one level).

### E-selectin

Eighteen studies investigated E-selectin in a total of 831 RA patients (mean age 53 years, 78% females) and 720 healthy controls (mean age 50 years, 72% females) [83, 84, 86, 88, 89, 91, 93, 95, 96, 99, 102–104, 106–109, 112]. Twelve studies were conducted in Europe [83, 84, 86, 88, 89, 91, 103, 104, 106, 108, 109, 112], 2 in America [99, 107], 2 in Asia [95, 102], and 2 in Africa [93, 96]. E-selectin was measured using an ELISA in all studies. Measurements were performed in serum in 13 studies [83, 84, 86, 88, 89, 91, 93, 95, 96, 99, 107–109] and plasma in 3 [102, 104, 112]. Details regarding the matrix assessed were missing in two studies [103, 106]. RA duration, reported in 12 studies, ranged between 0.25 and 23 years [84, 88, 89, 93, 95, 96, 99, 102, 103, 108, 109, 112], whereas the DAS-28 reported in 6 studies ranged between 3.6 and 6.23 [99, 103, 106, 108, 109, 112]. Twelve studies reported data on glucocorticoid treatment [84, 86, 88, 91, 99, 102–104, 106, 108, 109, 112] and 12 on DMARDs treatment [84, 86, 88, 89, 91, 93, 99, 102, 103, 106, 108, 109]. The risk of bias was low in seven studies [89, 93, 99, 104, 107, 109, 112], moderate in eight [83, 84, 86, 88, 91, 95, 96, 102], and high in the remaining three [103, 106, 108].

**Fig. 8:**
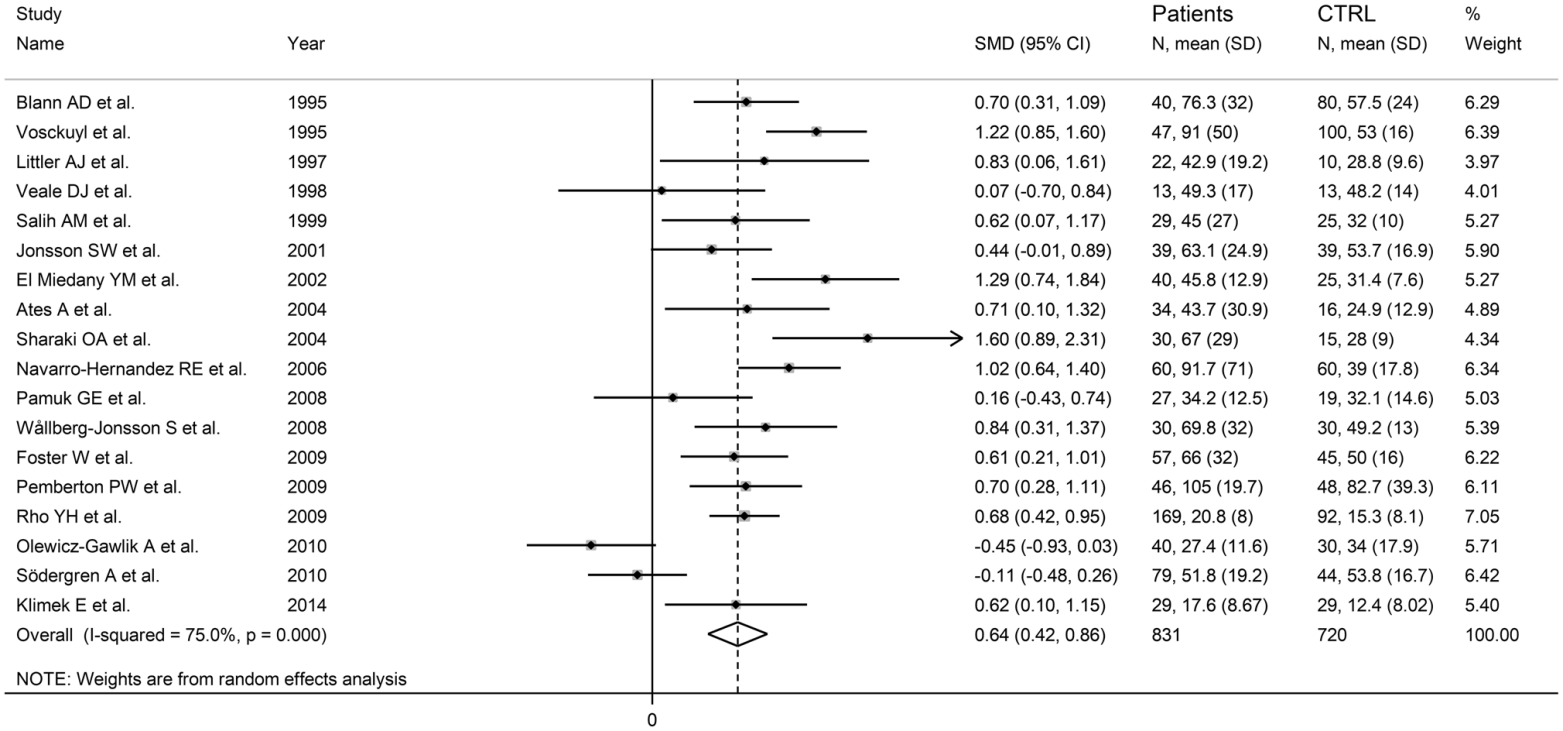
Forest plot of studies investigating E-selectin in RA patients and controls

The forest plot showed that E-selectin concentrations were significantly higher in RA patients when compared to controls (SMD = 0.64, 95% CI 0.42–0.86, *p* < 0.001; *I*^2^ = 75.0%, *p* < 0.001; Fig. 8). Sensitivity analysis showed that the pooled SMD values were stable, ranging between 0.59 and 0.70 (Supplementary Fig. S8). There was no significant publication bias with the Begg’s test (*p* = 0.82) or the Egger’s test (*p* = 0.98). The “trim-and-fill” method identify two missing studies to be added to the left side of the funnel plot to ensure symmetry (Supplementary Fig. S9). However, the resulting effect size remained significant (SMD = 0.56, 95% CI 0.33–0.78; *p* < 0.001).

**Fig. 9:**
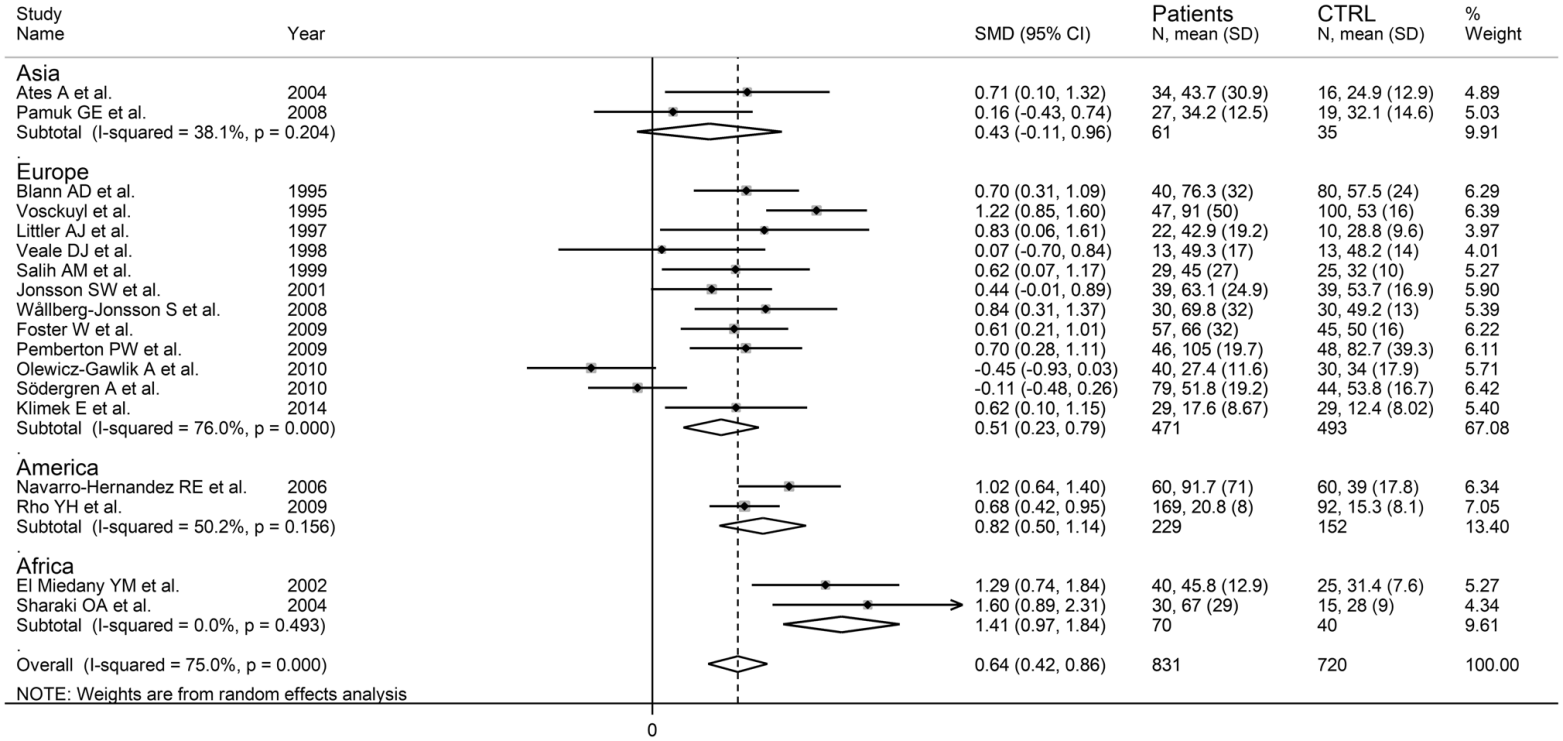
Forest plot of studies investigating E-selectin in RA patients and controls according to study continent

No significant associations were observed in meta-regression between the effect size and age (*t* = 0.10, *p* = 0.92), sex distribution (*t* = − 0.64, *p* = 0.54), publication year (*t* = − 1.54, *p* = 0.14), number of participants (*t* = 0.33, *p* = 0.75), CRP (*t* = 1.06, *p* = 0.31), ESR (*t* = 1.70, *p* = 0.12), RA duration (*t* = 0.83, *p* = 0.43), or use of DMARDs (*t* = − 0.54, *p* = 0.60) and glucocorticoids (*t* = − 0.87, *p* = 0.41). In subgroup analysis, there was a significant difference (*p* = 0.02) in pooled SMD between Asian (SMD = 0.43, 95% CI − 0.11 to 0.96, *p* = 0.12; *I*^2^ = 38.1%, *p* = 0.204), European (SMD = 0.51, 95% CI 0.23–0.79, *p* < 0.001; *I*^2^ = 76.0%, *p* < 0.001), American (SMD = 0.82, 95% CI 0.50–1.14, *p* < 0.001; *I*^2^ = 50.2%, *p* = 0.156), and African studies (SMD = 1.41, 95% CI 0.97–1.84, *p* < 0.001; *I*^2^ = 0.0%, *p* = 0.493; Fig. 9), with a virtually absent heterogeneity in the African subgroup. By contrast, there were non-significant differences (*p* = 0.61) in pooled SMD values between studies investigating serum (SMD = 0.65, 95% CI 0.36–0.95, *p* < 0.001; *I*^2^ = 81.4%, %, *p* < 0.001) and plasma (SMD = 0.51, 95% CI 0.23–0.79, *p* < 0.001; *I*^2^ = 0.0%, *p* = 0.405; Supplementary Fig. S10), with a virtually absent heterogeneity in the plasma subgroup.

The overall level of certainty remained low (rating 2) after considering the low–moderate risk of bias in most studies (no change), the moderate and partially explained heterogeneity (no change), the lack of indirectness (no change), the moderate effect size (SMD = 0.64, no change) [119], and the absence of publication bias (no change).

### L-selectin

Six studies, all conducted in Europe, investigated L-selectin in a total of 230 RA patients (mean age 51 years, 80% females) and 170 healthy controls (mean age 48 years, 81% females) [86, 90, 95, 103, 108, 109]. L-selectin was measured in serum by ELISA in all studies, except one study which did not provide relevant details regarding the matrix assessed [103]. The risk of bias was low in one study [109], moderate in three [86, 90, 95], and high in the remaining two [103, 108].

**Fig. 10:**
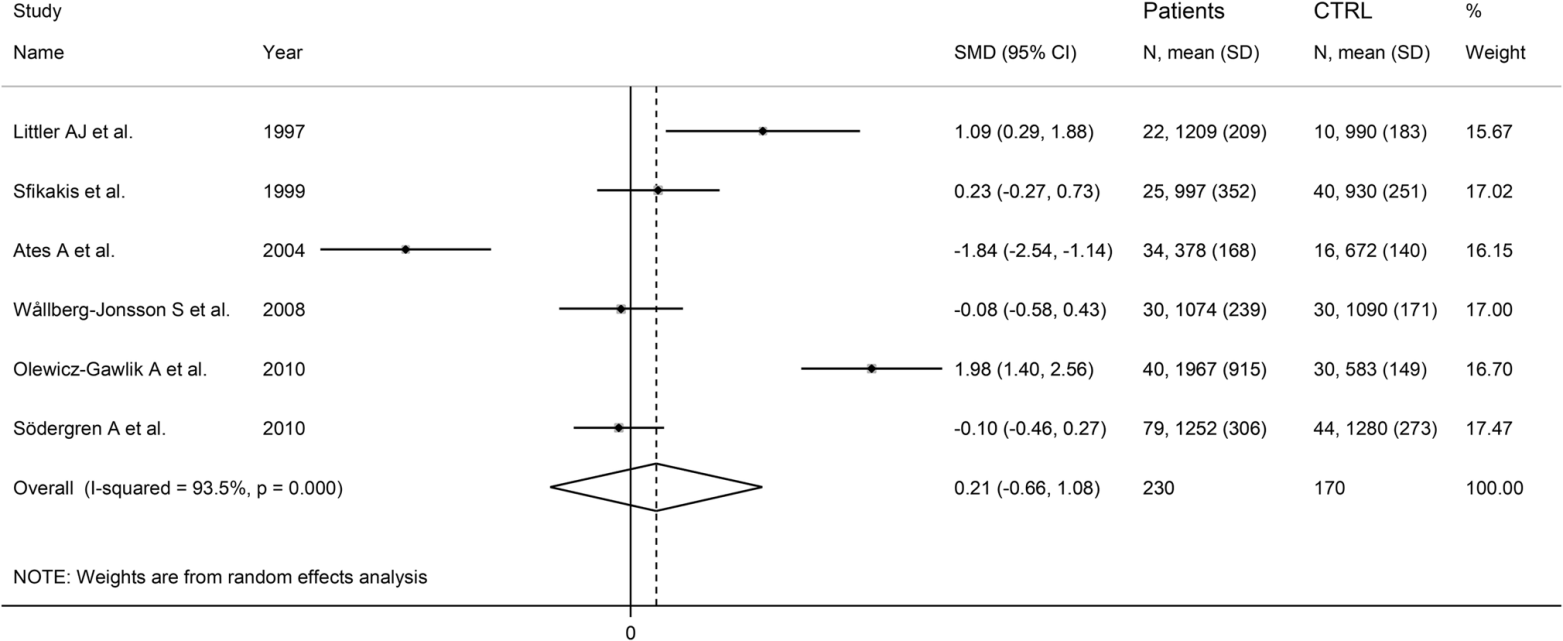
Forest plot of studies investigating L-selectin in RA patients and controls

The forest plot showed the absence of significant between-group differences in L-selectin concentrations (SMD = 0.21, 95% CI − 0.66 to 1.08, *p* = 0.63; *I*^2^ = 93.5%, *p* < 0.001; Fig. 10). Sensitivity analysis showed stability of the results, with pooled SMD values ranging between − 0.14 and 0.60 (Supplementary Fig. S11).

Assessment of publication bias, meta-regression and subgroup analysis could not be conducted because of the relatively small number of studies.

The overall level of certainty was downgraded to very low (rating 1) after considering the low–moderate risk of bias in most studies (no change), the high and unexplained heterogeneity (downgrade one level), the lack of indirectness (no change), the small effect size (SMD = 0.21, no change) [119], and the lack of assessment of publication bias (downgrade one level).

### P-selectin

Eight studies investigated P-selectin in a total of 230 RA patients (mean age 53 years, 85% females) and 211 healthy controls (mean age 51 years, 87% females) [86–88, 90, 95, 103, 106, 108]. Six studies were conducted in Europe [86, 88, 90, 103, 106, 108], and two in Asia [87, 95]. P-selectin was measured using an ELISA assay in serum in four studies [86, 90, 95, 108] and in plasma in two studies [87, 88]. Two studies did not report relevant information regarding the matrix analyzed [103, 106]. The risk of bias was moderate in five studies [86–88, 90, 95] and high in the other three [103, 106, 108] (Table 2).

**Fig. 11:**
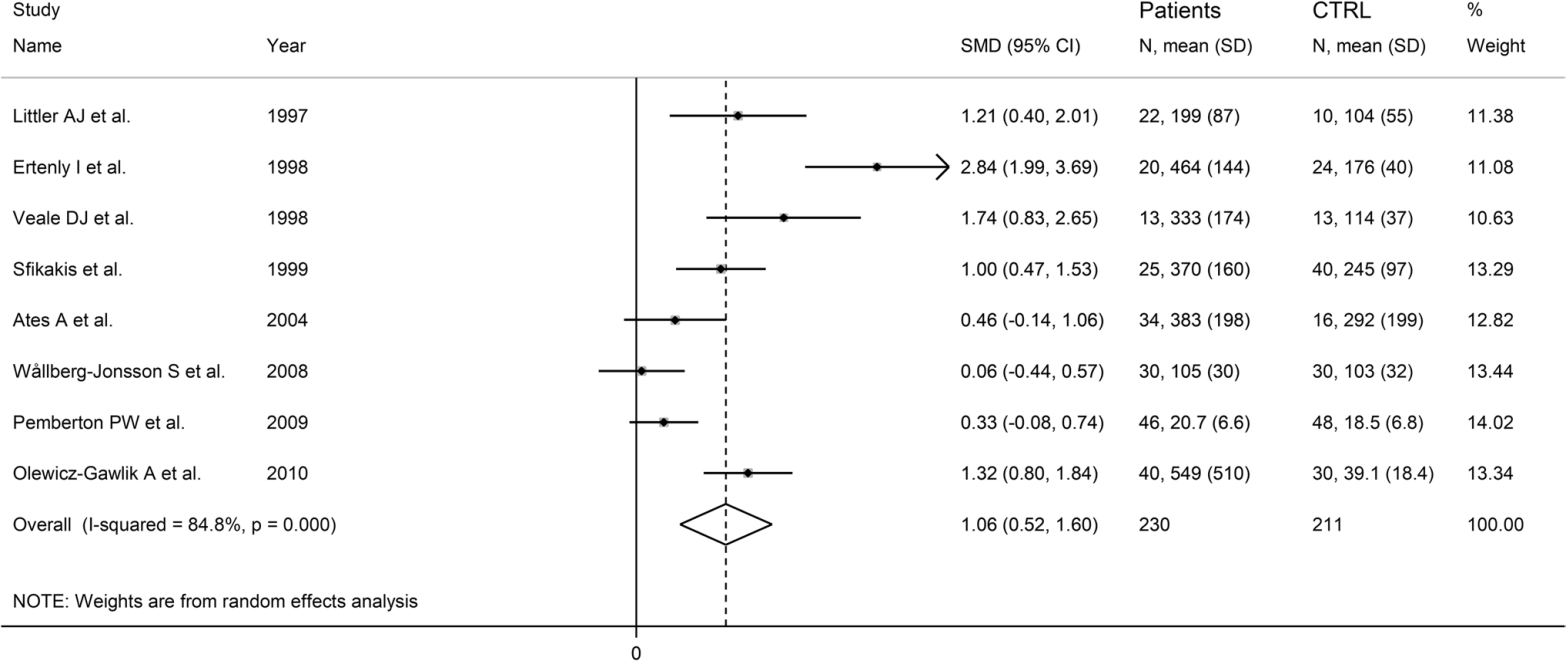
Forest plot of studies investigating P-selectin in RA patients and controls

The forest plot showed that P-selectin concentrations were significantly higher in RA patients when compared to controls (SMD = 1.06, 95% CI 0.50–1.60, *p* < 0.001; *I*^2^ = 84.8%, *p* < 0.001; Fig. 11). The pooled SMD values were stable in sensitivity analysis and ranged between 0.81 and 1.21 (Supplementary Fig. S12). Assessment of publication bias and meta-regression analysis could not be performed because of the limited number of studies.

**Fig. 12:**
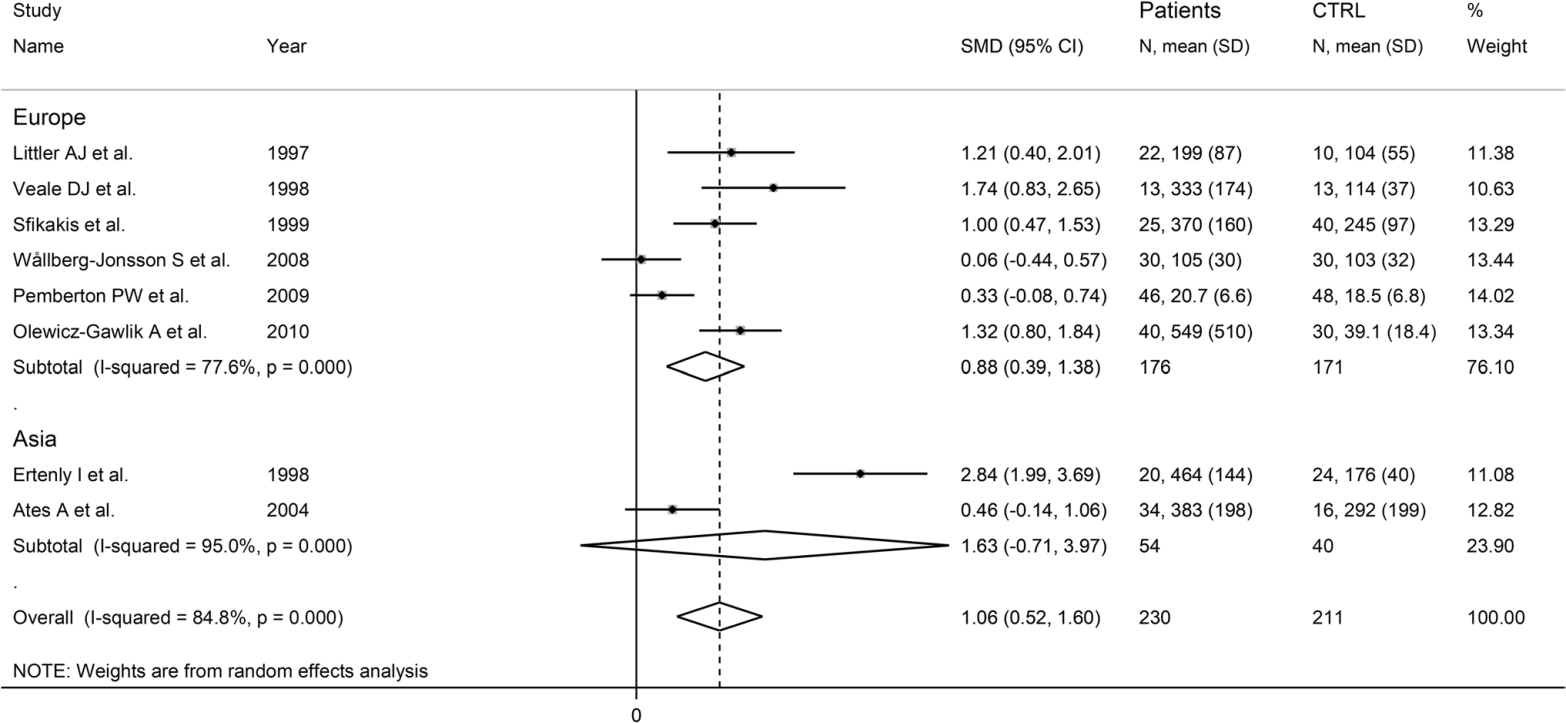
Forest plot of studies investigating P-selectin in RA patients and controls according to study continent

**Fig. 13:**
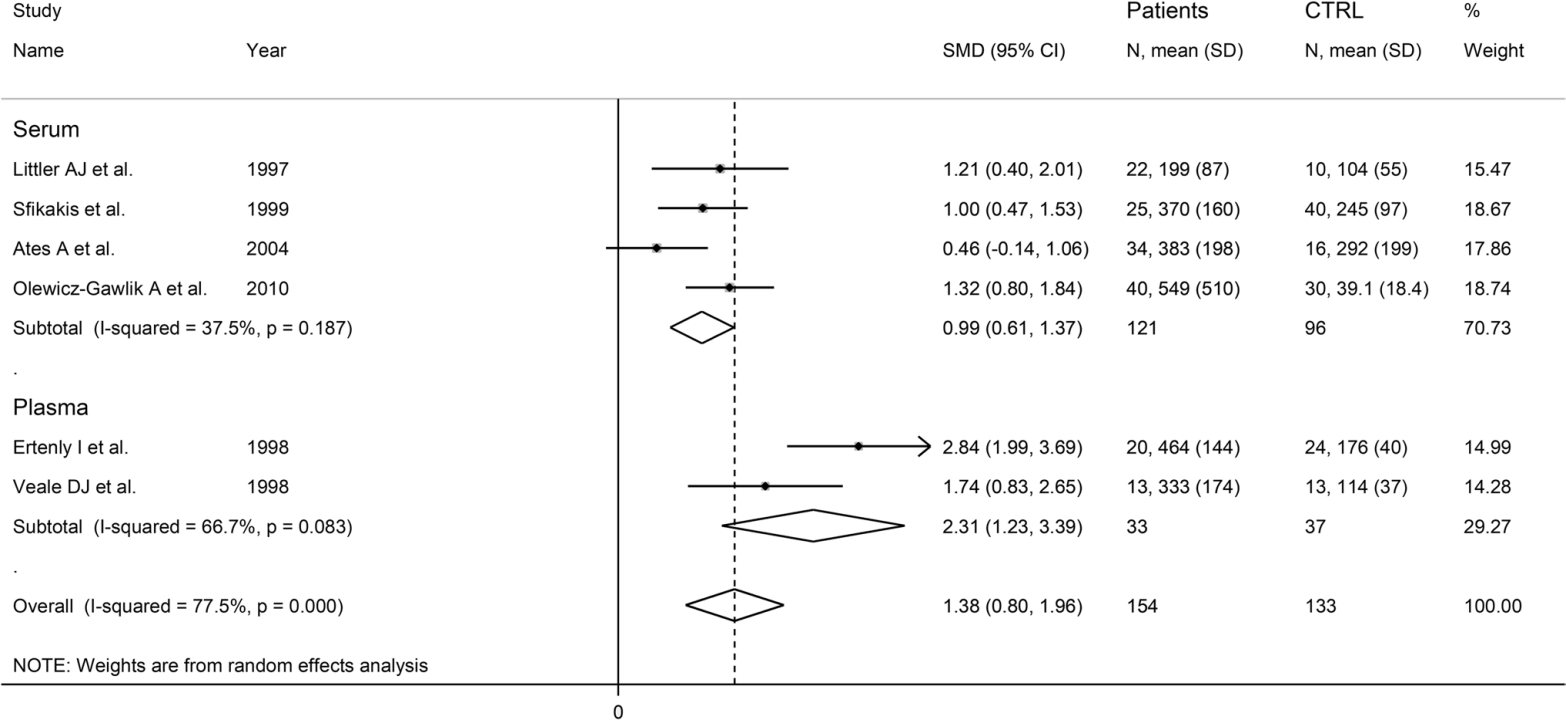
Forest plot of studies investigating P-selectin in RA patients and controls according to the type of matrix assessed (serum or plasma)

In subgroup analysis, the pooled SMD was significantly different in European studies (SMD = 0.88, 95% CI 0.39–1.38, *p* < 0.001; *I*^2^ = 77.6%, *p* < 0.001), but not in Asian studies (SMD = 1.63, 95% CI − 0.71 to 3.97, *p* = 0.17; *I*^2^ = 95.0%, *p* < 0.001; Fig. 12). A significant difference (*p* = 0.045) in pooled SMD was observed between studies on serum (SMD = 0.99, 95% CI 0.61–1.37, *p* < 0.001; *I*^2^ = 37.5%, *p* = 0.187), and those on plasma (SMD = 2.31, 95% CI 1.23–3.39, *p* < 0.001; *I*^2^ = 66.7%, *p* = 0.083, Fig. 13), with a decreased between-study variance in the serum subgroup.

The overall level of certainty remained low (rating 2) after considering the moderate risk of bias in most studies (no change), the high but partially explained heterogeneity (no change), the lack of indirectness (no change), the large effect size (SMD = 1.06, upgrade one level) [119], and the lack of assessment of publication bias (downgrade one level).

## Discussion

Our study supports a significant pathophysiological role of cell adhesion molecules in mediating the interplay between RA and atherosclerosis. Specifically, RA patients had significantly higher concentrations of VCAM-1, ICAM-1, E-selectin, and P-selectin, but not L-selectin, when compared to healthy controls. The corresponding pooled SMD values were stable in sensitivity analysis. In meta-regression and subgroup analysis, we observed significant associations between the effect size of ICAM-1 and the use of glucocorticoids, between the effect size of VCAM-1 and ERS, between the effect size of VCAM-1, E-selectin, and P-selectin and study continent, and between the effect size of P-selectin and the type of matrix assessed (plasma vs. serum).

The atherosclerotic process is characterized by a significant dysregulation of inflammatory and immune cellular and molecular pathways [120, 121]. As part of such dysregulation, the abnormal interaction between leukocytes and endothelial cells, mediated by cell adhesion molecules, plays a critical role in the early stages of plaque formation [122]. The consequent accumulation of leukocytes in the intima layer, in turn, facilitates their uptake of lipoprotein particles and subsequent differentiation into macrophages, precursors of foam cells, critically involved in plaque growth [123–125]. Accordingly, several studies have reported the upregulation of immunoglobulin-like adhesion molecules and selectins in experimental models of atherosclerosis [55, 126–129].

The significant elevations in VCAM-1, ICAM-1, E-selectin, and P-selectin in RA patients observed in our systematic review and meta-analysis reflect a state of endothelial activation and dysregulation, in the context of excessive inflammation and oxidative stress. Furthermore, they suggest the potential utility of measuring cell adhesion molecules for cardiovascular risk stratification in this patient group. The lack of significant between-group differences in the concentrations of L-selectin, a critical regulator of leukocyte tethering, rolling, adhesion, migration and signaling and monocyte protrusion during trans-endothelial migration [58, 130], indicates a different pathophysiological role of this selectin in RA. However, the relatively small number of studies investigating L-selectin warrant further research to confirm this proposition. Furthermore, the lack of significant associations in meta-regression analysis between the effect size of the between-group differences in cell adhesion molecules and RA duration or DAS-28 also indicates that alterations in cell adhesion molecules are already present in patients with short disease duration and relatively low disease activity.

Another interesting observation was the significant inverse association between the effect size of ICAM-1 and the use of glucocorticoids. This finding is in line with the results of in vitro studies reporting that dexamethasone, a potent glucocorticoid, inhibits the expression of E-selectin and ICAM-1 in endothelial cells following treatment with the endotoxin lipopolysaccharide, a known stimulator of acute pro-inflammatory responses [131]. Other studies have reported a similar effect of glucocorticoids on the expression of cell adhesion molecules [132–134]. Accordingly, the observation of a significant positive association between the effect size of VCAM-1 and ESR support the traditional proposition that endothelial activation and dysregulation are intimately linked with excess inflammation [135–137]. However, as such associations were observed with specific cell adhesion molecules, i.e., ICAM-1 and VCAM-1, further research is warranted to confirm these findings in patients with RA.

In subgroup analysis, a significant association was observed the SMD of VCAM-1, E-selectin, and P-selectin and study continent, suggesting the presence of ethnic-related differences in cell adhesion molecules. Specifically, the SMD of VCAM-1 was progressively higher in American, European, and African studies, the SMD of E-selectin was progressively higher in Asian, European, American, and African studies, and the SMD of P-selectin was significant in European but not Asian studies. In an epidemiological study conducted in England, participants of African background had significantly lower concentrations of VCAM-1 and ICAM-1 when compared to Caucasian and South Asian participants [138]. Relatively higher VCAM-1 concentrations in Caucasians vs. African Americans, Hispanics, and Chinese participants have also been reported in a North American study [139]. By contrast, studies have generally failed to identify the presence of significant ethnic-related differences in circulating selectins [140–143]. It is important to emphasize however that these studies generally investigated patients with relatively low cardiovascular risk and without autoimmune diseases, suggesting that additional studies are required to investigate possible ethnic-related differences in the concentrations of cell adhesion molecules in patients with RA and other rheumatic diseases.

Strengths of our systematic review and meta-analysis include the combined assessment of a range of immunoglobulin-like adhesion molecules and selectins in patients with RA in a relatively large number of studies, and the robust assessment of the risk of bias and the certainty of evidence for each adhesion molecule. A possible limitation is related to the high heterogeneity observed for the studied adhesion molecules. However, specific sources of heterogeneity were identified for ICAM-1 (study continent and matrix assessed), VCAM-1 (matrix assessed), E-selectin (study continent and matrix assessed), and P-selectin (study continent).

In conclusion, our systematic review and meta-analysis suggests that cell adhesion molecules play an important pathophysiological role in the interplay between RA and atherosclerosis, including patients with relatively short RA duration and low disease activity. Further studies are warranted to investigate the potential utility of cell adhesion molecules in cardiovascular risk stratification and the possible effects of immunomodulatory and anti-inflammatory treatments.

### Supplementary Information

Below is the link to the electronic supplementary material.

Supplementary file 1 (TIF 4758 kb)

Supplementary file 2 (TIF 1336 kb)

Supplementary file 3 (TIF 4438 kb)

Supplementary file 4 (docx 13 kb)

Supplementary file 5 (TIF 4278 kb)

Supplementary file 6 (TIF 1338 kb)

Supplementary file 7 (TIF 3573 kb)

Supplementary file 8 (TIF 4019 kb)

Supplementary file 9 (TIF 1363 kb)

Supplementary file 10 (TIF 3278 kb)

Supplementary file 11 (TIF 2485 kb)

Supplementary file 12 (TIF 2774 kb)

Supplementary file 13 (DOCX 12 kb)

Supplementary file 14 (DOCX 19 kb)

Supplementary file 15 (TIF 5 mb)

## Data Availability

The data that support the findings of this systematic review and meta-analysis are available from AZ upon reasonable request.
