# Supplementary figures and images for: A systematic review and meta-analysis of circulating adhesion molecules in rheumatoid arthritis
Source: Inflamm Res. 2024 Jan 19;73(3):305–27. doi: 10.1007/s00011-023-01837-6 (PMC10894129; doi:10.1007/s00011-023-01837-6)

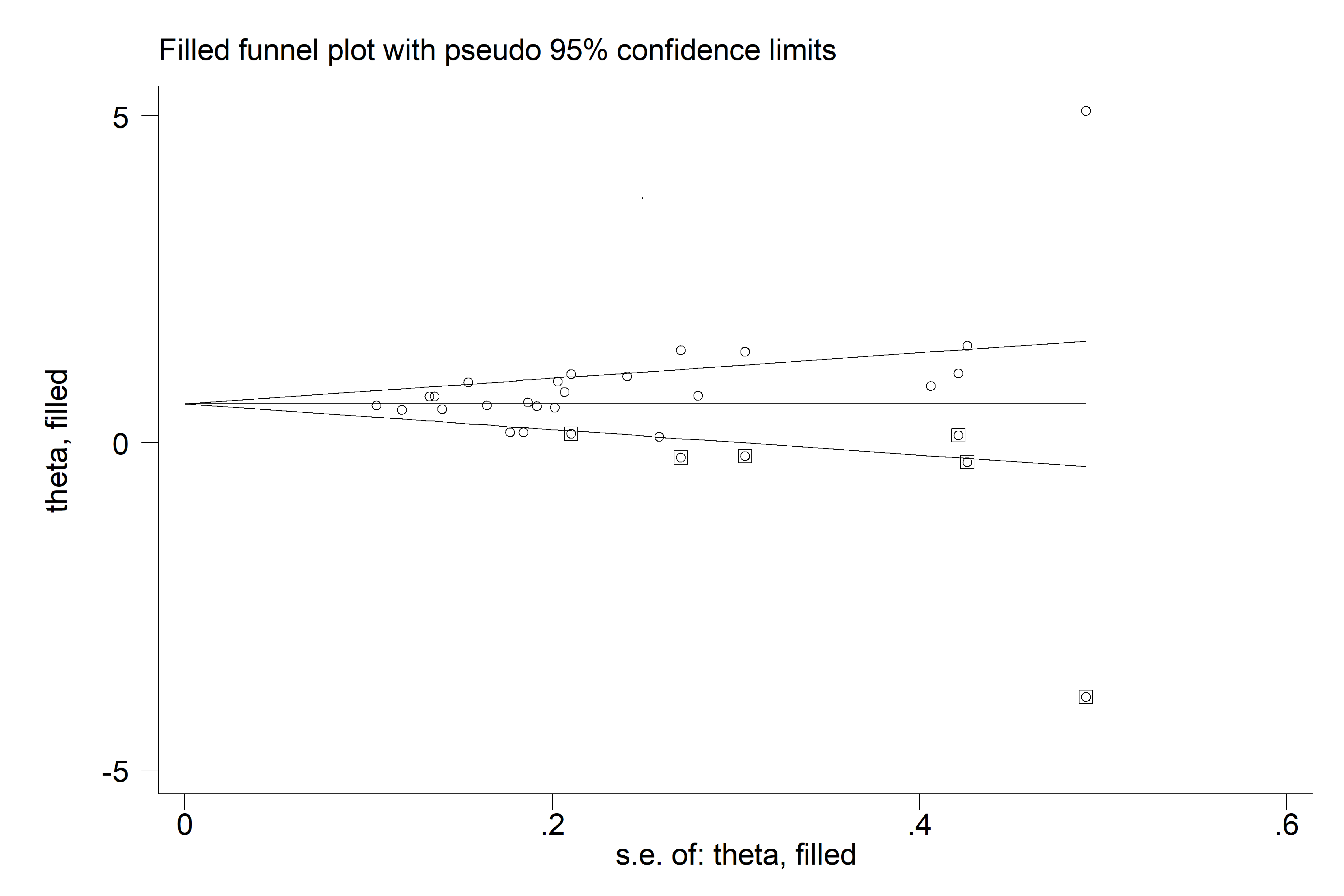

Supplement: Supplementary file 1 — (TIF 4758 kb) [file 11_2023_1837_MOESM1_ESM.tif]

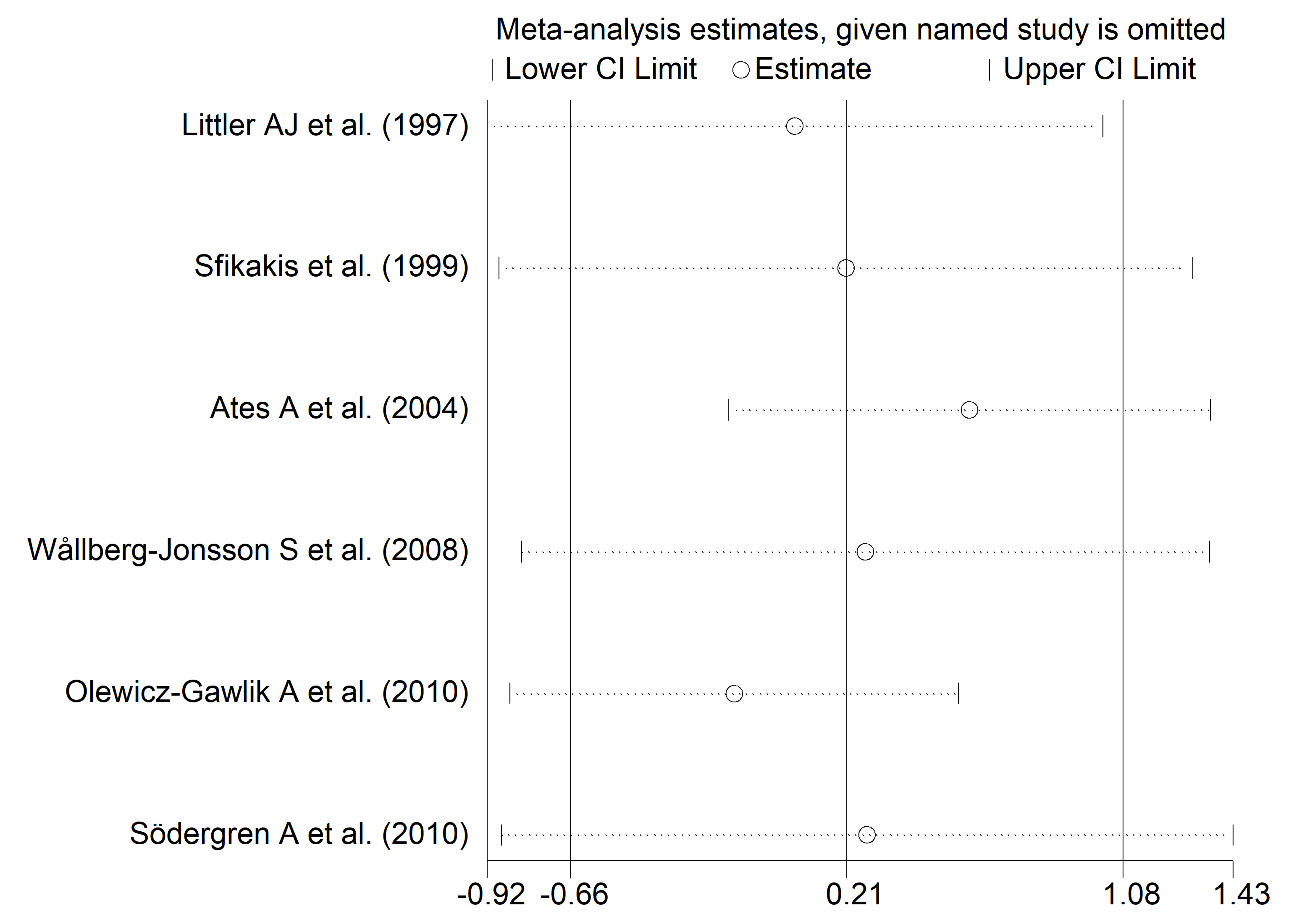

Supplement: Supplementary file 2 — (TIF 1336 kb) [file 11_2023_1837_MOESM2_ESM.tif]

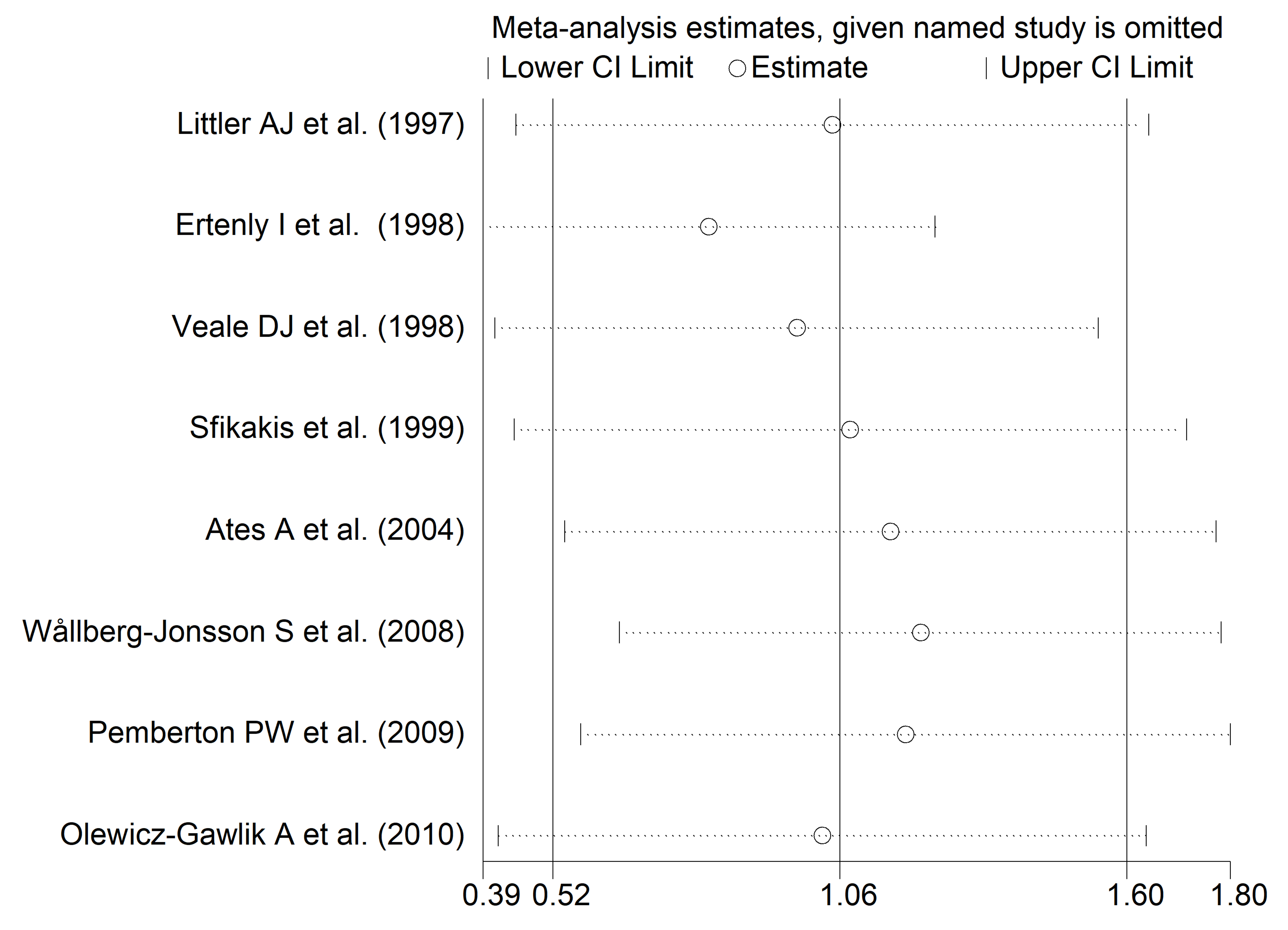

Supplement: Supplementary file 3 — (TIF 4438 kb) [file 11_2023_1837_MOESM3_ESM.tif]

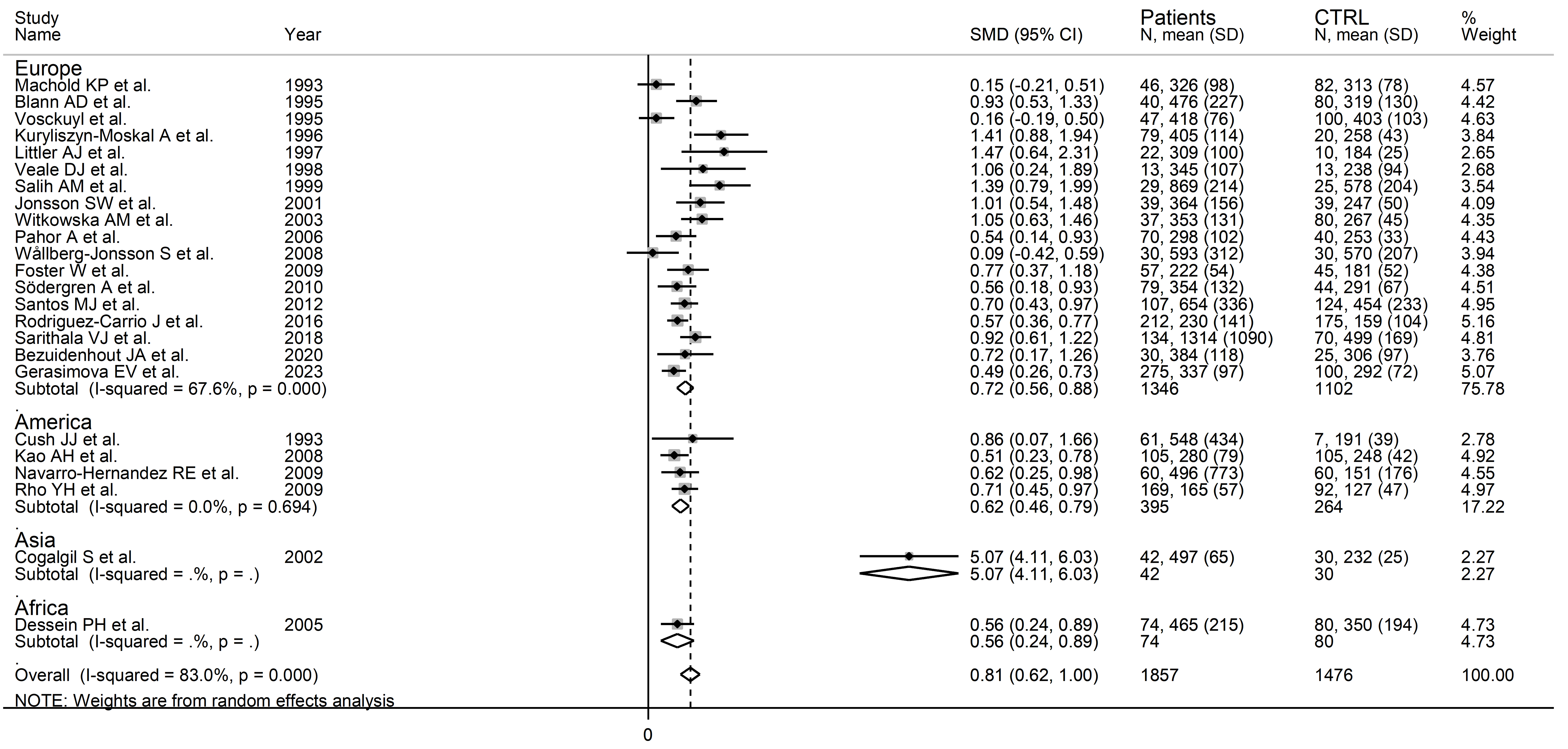

Supplement: Supplementary file 5 — (TIF 4278 kb) [file 11_2023_1837_MOESM5_ESM.tif]

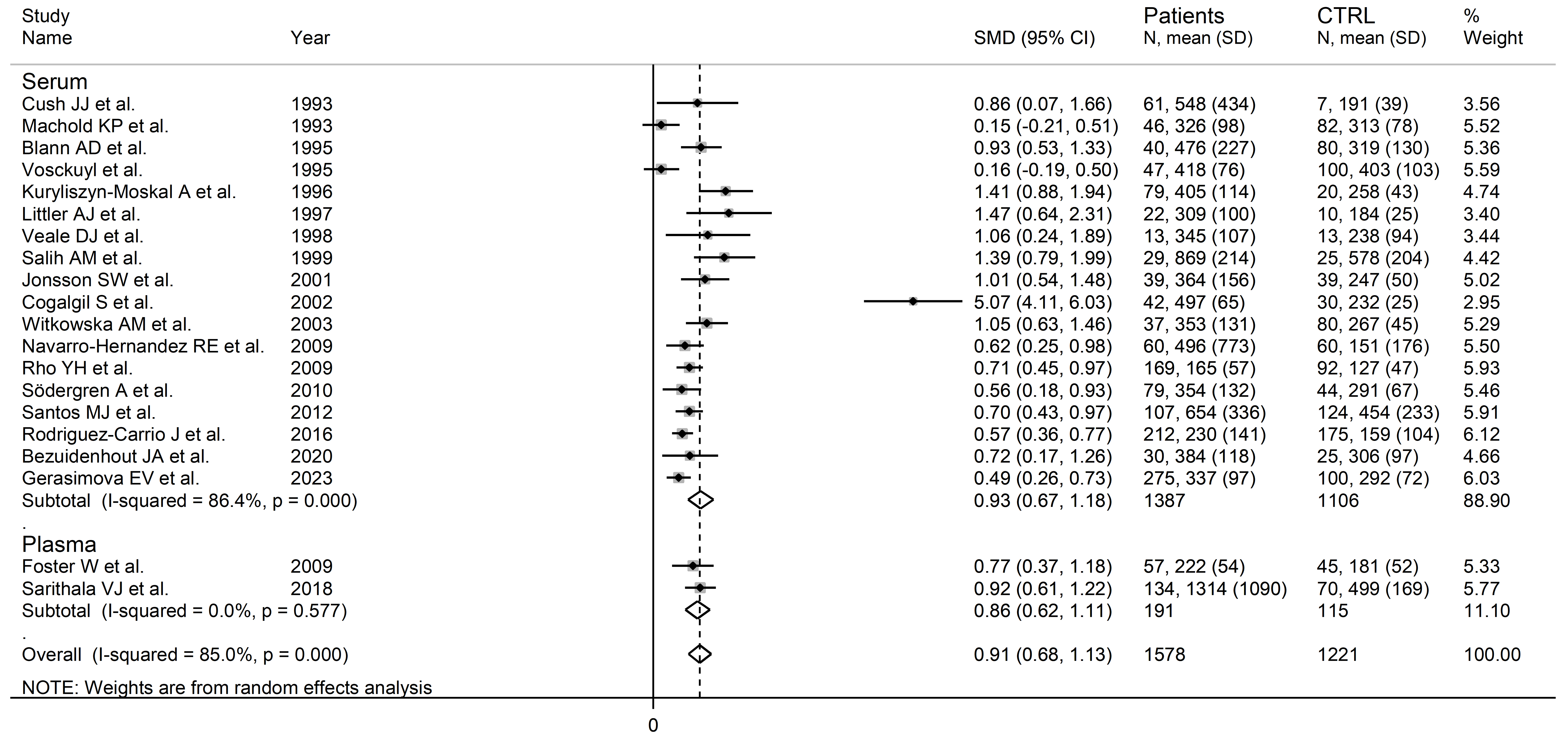

Supplement: Supplementary file 6 — (TIF 1338 kb) [file 11_2023_1837_MOESM6_ESM.tif]

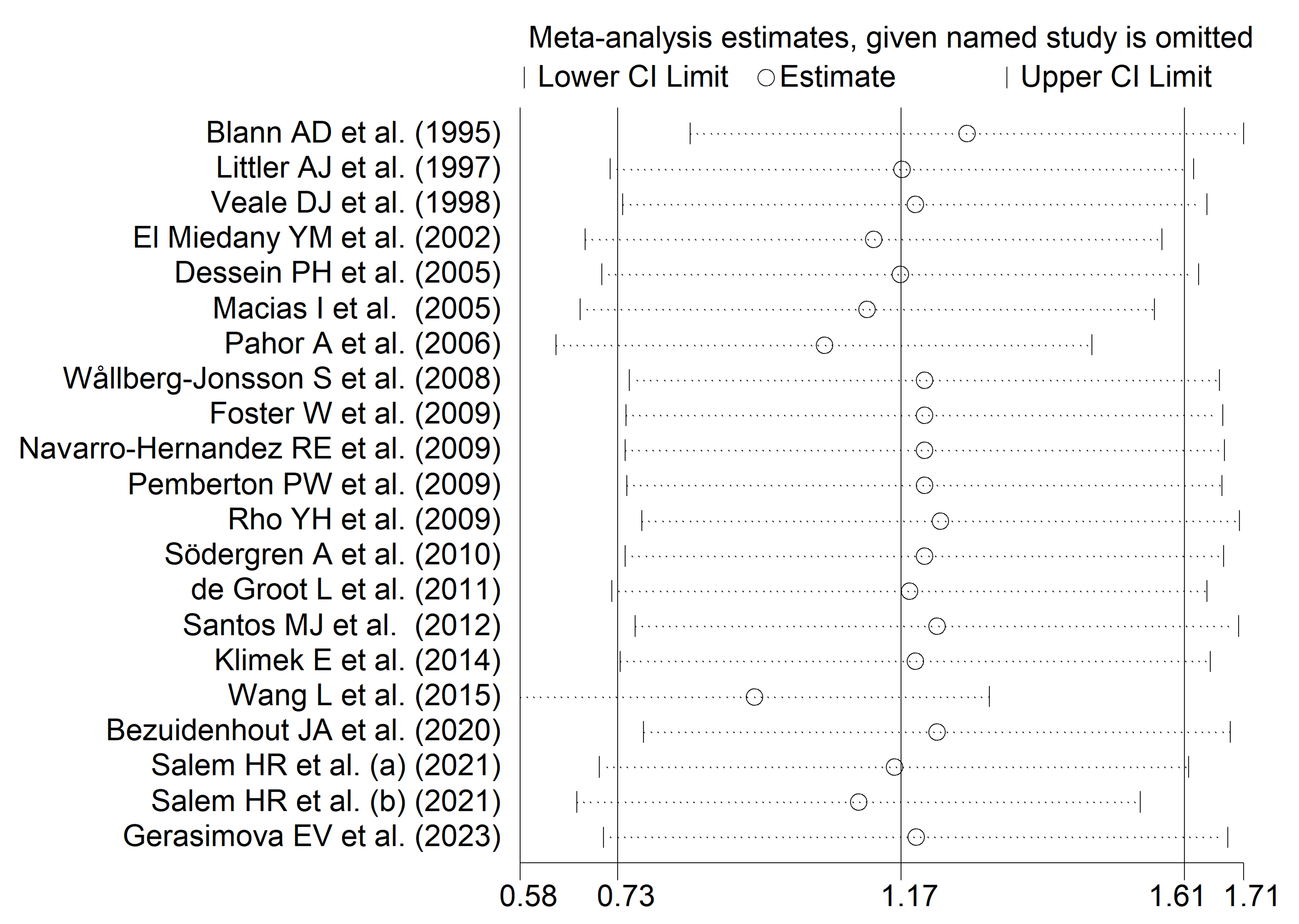

Supplement: Supplementary file 7 — (TIF 3573 kb) [file 11_2023_1837_MOESM7_ESM.tif]

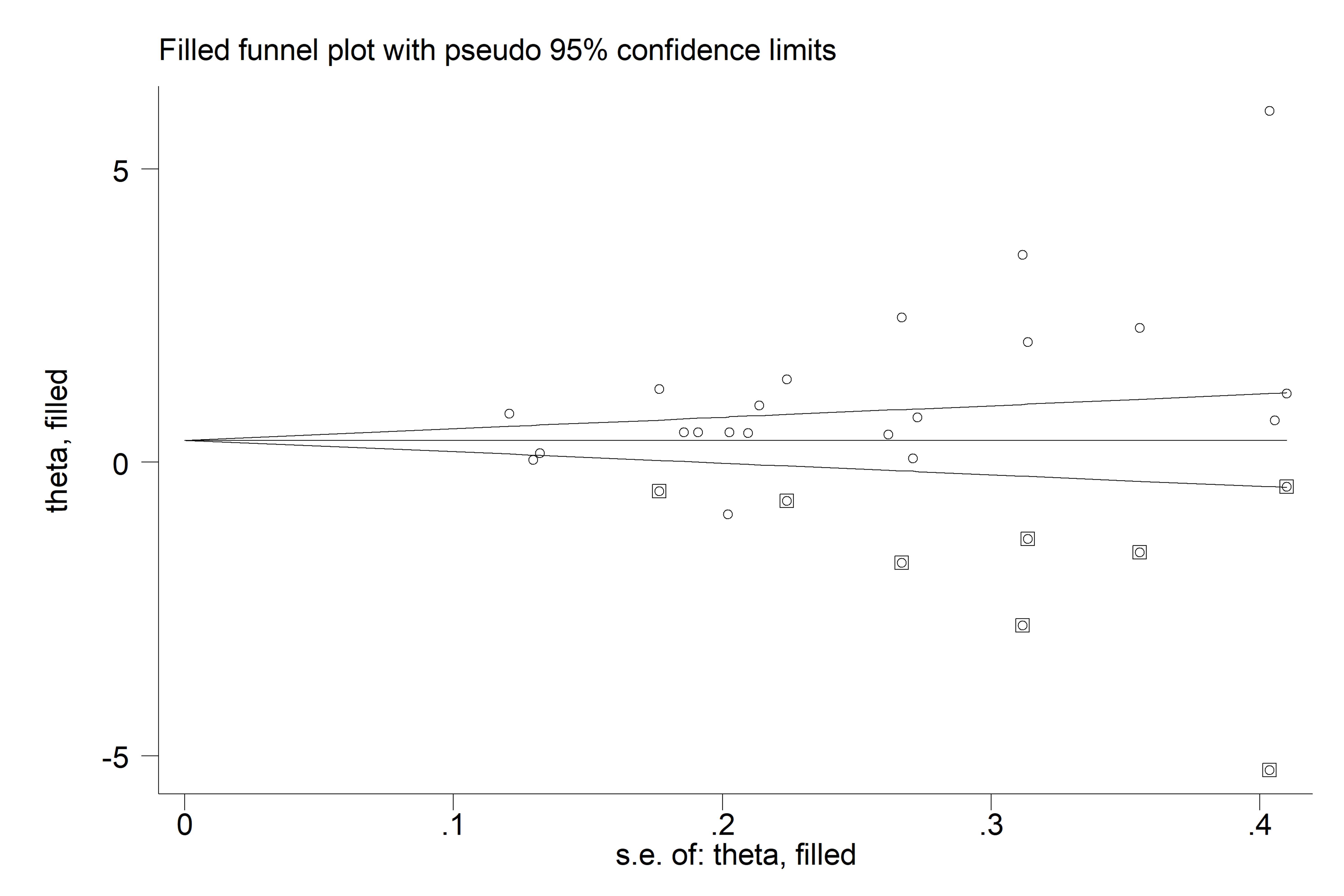

Supplement: Supplementary file 8 — (TIF 4019 kb) [file 11_2023_1837_MOESM8_ESM.tif]

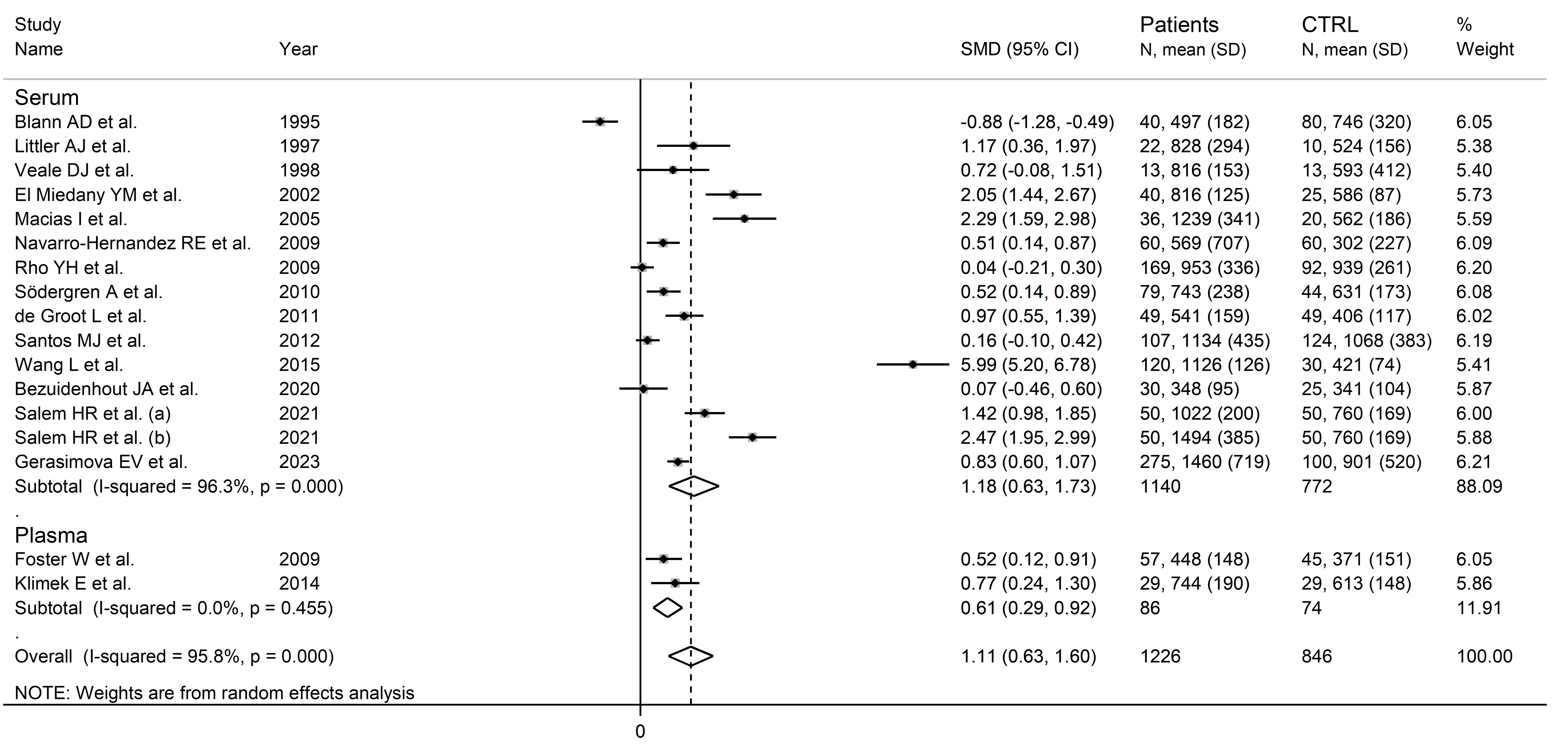

Supplement: Supplementary file 9 — (TIF 1363 kb) [file 11_2023_1837_MOESM9_ESM.tif]

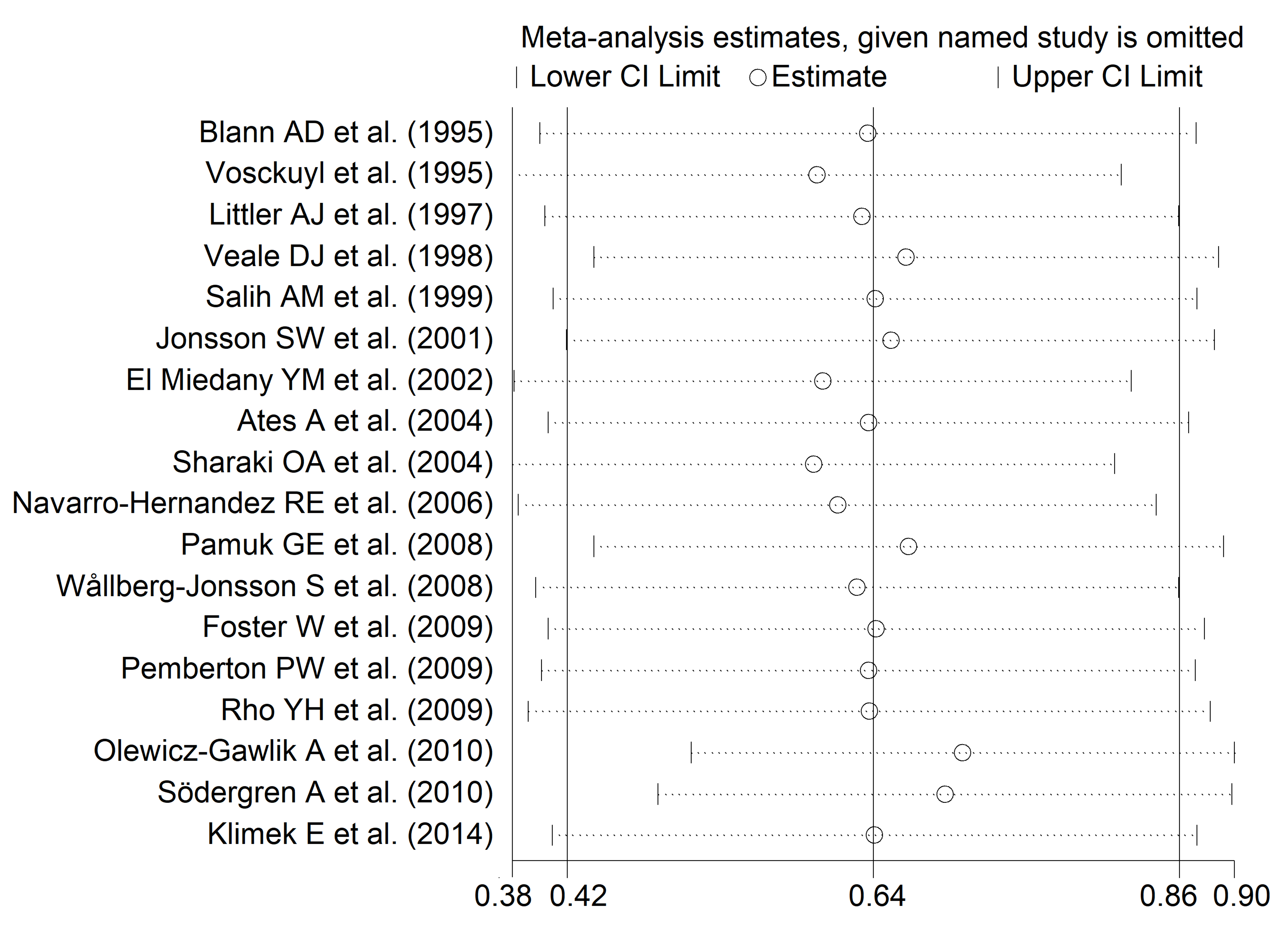

Supplement: Supplementary file 10 — (TIF 3278 kb) [file 11_2023_1837_MOESM10_ESM.tif]

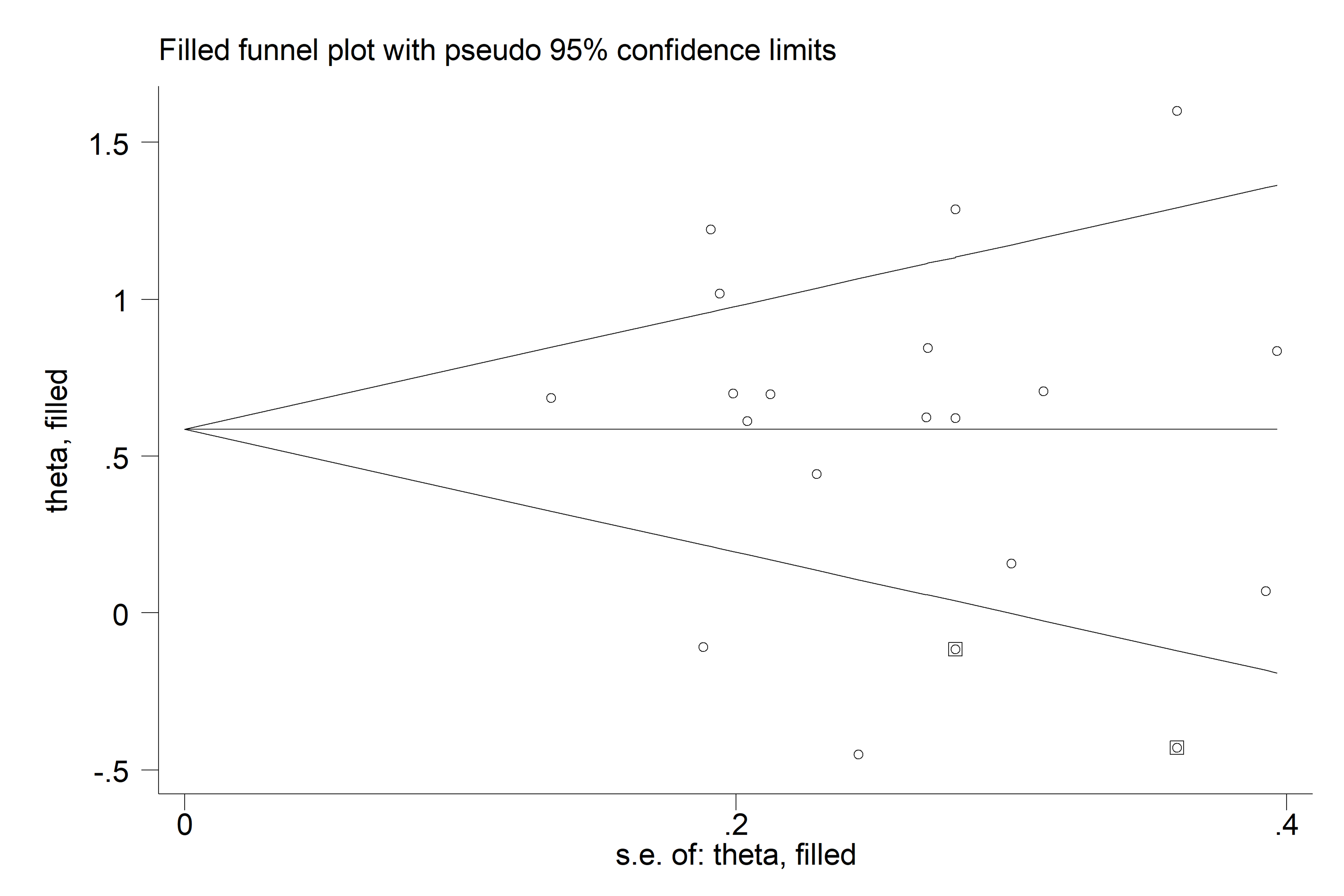

Supplement: Supplementary file 11 — (TIF 2485 kb) [file 11_2023_1837_MOESM11_ESM.tif]

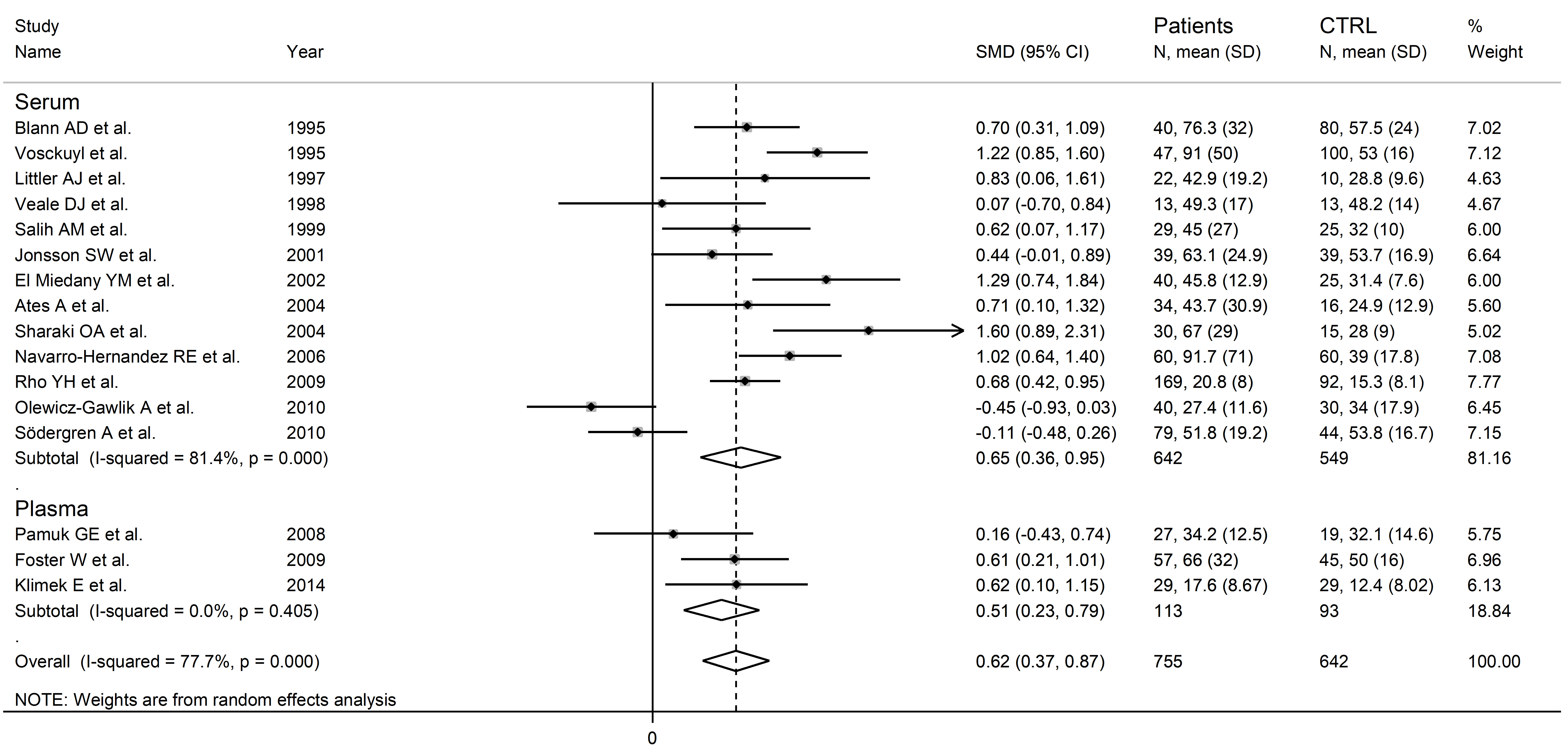

Supplement: Supplementary file 12 — (TIF 2774 kb) [file 11_2023_1837_MOESM12_ESM.tif]

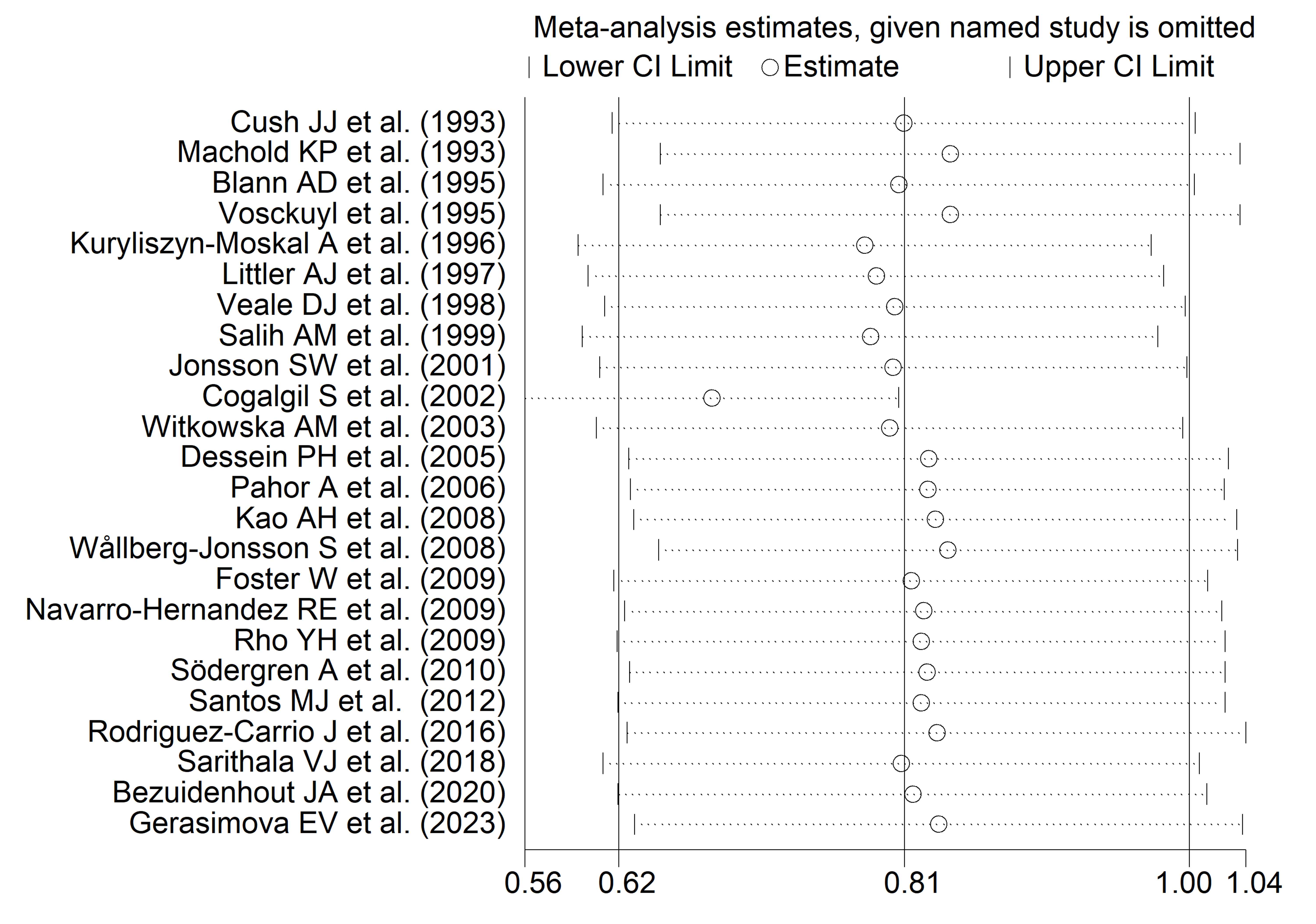

Supplement: Supplementary file 15 — (TIF 5 mb) [file 11_2023_1837_MOESM15_ESM.tif]
