# Supplementary material for: A systematic review and meta-analysis of circulating adhesion molecules in rheumatoid arthritis
Source: Inflamm Res. 2024 Jan 19;73(3):305–27. doi: 10.1007/s00011-023-01837-6 (PMC10894129; doi:10.1007/s00011-023-01837-6)
Supplement: Supplementary file 4 — (docx 13 kb) [file 11_2023_1837_MOESM4_ESM.docx]

**Supplementary figure legends**

**Supplementary Figure 1.** Sensitivity analysis of the association between ICAM-1 and RA.

**Supplementary Figure 2.** Funnel plot of studies investigating the association between ICAM-1 and RA after “trimming-and-filling.” Dummy studies and genuine studies are represented by enclosed circles and free circles, respectively.

**Supplementary Figure 3.** Forest plot of studies investigating ICAM-1 in RA patients and controls according to study continent.

**Supplementary Figure 4.** Forest plot of studies investigating ICAM-1 in RA patients and controls according to the matrix investigated (serum or plasma).

**Supplementary Figure 5.** Sensitivity analysis of the association between VCAM-1 and RA.

**Supplementary Figure 6.** Funnel plot of studies investigating the association between VCAM-1 and RA after “trimming-and-filling.” Dummy studies and genuine studies are represented by enclosed circles and free circles, respectively.

**Supplementary Figure 7.** Forest plot of studies investigating VCAM-1 in RA patients and controls according to the type of matrix assessed (serum or plasma).

**Supplementary Figure 8.** Sensitivity analysis of the association between E-selectin and RA.

**Supplementary Figure 9.** Funnel plot of studies investigating the association between E-selectin and RA after “trimming-and-filling.” Dummy studies and genuine studies are represented by enclosed circles and free circles, respectively.

**Supplementary Figure 10.** Forest plot of studies investigating E-selectin in RA patients and controls according to the type of matrix assessed (serum or plasma).

**Supplementary Figure 11.** Sensitivity analysis of the association between L-selectin values and RA.

**Supplementary Figure 12.** Sensitivity analysis of the association between P-selectin values and RA**.**
